# Distribution and phylogenetics of whiteflies and their endosymbiont relationships after the Mediterranean species invasion in Brazil

**DOI:** 10.1038/s41598-018-32913-1

**Published:** 2018-10-01

**Authors:** Letícia Aparecida de Moraes, Cristiane Muller, Regiane Cristina Oliveira de Freitas Bueno, Antônio Santos, Vinicius Henrique Bello, Bruno Rossitto De Marchi, Luís Fernando Maranho Watanabe, Julio Massaharu Marubayashi, Beatriz Rosa Santos, Valdir Atsushi Yuki, Hélio Minoru Takada, Danielle Ribeiro de Barros, Carolina Garcia Neves, Fábio Nascimento da Silva, Mayra Juline Gonçalves, Murad Ghanim, Laura Boykin, Marcelo Agenor Pavan, Renate Krause-Sakate

**Affiliations:** 1 São Paulo State University, UNESP-FCA, Department of Plant Protection, CEP, 18610-034 Botucatu, (SP) Brazil; 2 Corteva Agriscience, 13801-540 Mogi-Mirim, (SP) Brazil; 3 Instituto Agronômico de Campinas, CEP, 13075-630 Campinas, (SP) Brazil; 4 Universidade Federal de Pelotas, Department of Plant Protection, CEP, 96010-610 Pelotas, (RS) Brazil; 5 Santa Catarina State University UDESC, Department of Agronomy/Plant Pathology, 88520-000 Lages, (SC) Brazil; 6 Institute of Plant Protection, Department of Entomology, The Volcani Center, Rishon LeZion, Israel; 7 The University of Western Australia, ARC Centre of Excellence in Plant Energy Biology and School of Chemistry and Biochemistry, Crawley, Perth, 6009 Western Australia Australia

## Abstract

The *Bemisia tabaci* is a polyphagous insect and a successful vector of plant viruses. *B. tabaci* is a species complex and in Brazil native species from the New World (NW) group, as well as the invasive species, Middle East-Asia Minor 1 (MEAM1) and Mediterranean (MED) were reported. For better understanding the distribution of the different species four years after the Mediterranean species invasion in Brazil, whiteflies were collected from 237 locations throughout the country between the years of 2013 and 2017, species were identified and the facultative endosymbionts detected. The survey revealed that MEAM1 was the prevalent species found on major crops across Brazil. It is the only species present in North, Northwestern and Central Brazil and was associated with virus-infected plants. MED was found in five States from Southeast to South regions, infesting mainly ornamental plants and was not associated with virus-infected plants. The prevalent endosymbionts identified in MEAM1 were *Hamiltonella* and *Rickettsia*; and the mtCOI analysis revealed low genetic diversity for MEAM1. In contrast, several different endosymbionts were identified in MED including *Hamiltonella*, *Rickettsia*, *Wolbachia* and *Arsenophonus;* and two distinct genetic groups were found based on the mtCOI analysis. Monitoring the distribution of the whiteflies species in Brazil is essential for proper management of this pest.

## Introduction

Over the past two decades, one of the most highlighted agricultural issue has been the emergence of *Bemisia tabaci* (Gennadius) (Hemiptera: Aleyrodidae) as a polyphagous phloem-feeder colonizing more than 600 host plants^1^ and being a successful vector that transmits over 300 plant virus species^2^. *B. tabaci* is a cryptic species complex composed of ecologically and genetically distinct groups, differentiated on the basis of biochemical or molecular polymorphism markers, and in characteristics such as the range of host plants utilized, the capacity to cause plant disorders, attraction of natural enemies, response to pesticides and plant virus transmission capabilities^3–6^. Yet, no morphological markers in any developmental stage of the life cycle can be used to differentiate between *B. tabaci* species^7–10^.

Recently, important progress has been made at the taxonomic level, with the current definition of at least 43 species based on the analysis of the mitochondrial cytochrome oxidase subunit I (mtCOI) gene^10–13^. The Middle East-Asia Minor 1 (MEAM1, formerly known as B biotype) and Mediterranean (MED, also known as Q biotype) species are considered the most invasive within this complex. However, the most likely factors that may have favored their worldwide expansion are likely related to their wide range of host plants and the global trade with plant materials that are suitable hosts for whiteflies^14,15^. Furthermore, invasiveness of *B. tabaci* might depend on the phenotype of inherited bacterial endosymbionts^16^ whose functions are not fully understood^10,17^. Examples of these roles include enhancing insecticide susceptibility^18,19^, facilitating virus transmission^20,21^ and conferring tolerance to high-temperature^22^. The obligatory endosymbiont *Portiera aleyrodidarum* is essential for the insect survival because it synthesizes several amino acids and carotenoids that the whitefly is unable to produce^17,23^. Besides, *B. tabaci* may harbor the facultative endosymbionts *Arsenophonus*, *Hamiltonella*, *Wolbachia, Cardinium*, *Fritschea*, *Rickettsia* and *Orientia*-like organism OLO^10,17,24^.

In Brazil, *B. tabaci* was first reported in 1928 in Bahia State^25^. The indigenous *B. tabaci* populations have driven outbreaks of bean golden mosaic diseases in common beans [*Phaseolus vulgaris* (Linnaeus) (Fabaceae)] in Brazil since the emergence of the virus in the 1960s. An extensive infection of weeds with a diversity of begomoviruses was also reported at that time^26,27^. Presumably, these outbreaks were caused by *B. tabaci* from the New World group (former A biotype), native from the Americas. Subsequently, two indigenous species, New World 1 (NW1) and New World 2 (NW2) were reported and identified in Brazil^12^. However, great concern with whiteflies in Brazil began in the 1990’s, after the introduction of the MEAM1 species in São Paulo State^28^. The tomato [*Solanum lycopersicum* Linnaeus (Solanaceae)] crop was severely affected by MEAM1 invasion with reports of at least 14 distinct indigenous tomato-infecting begomoviruses emerging locally following the introduction of MEAM1 in Brazil^27^. Two decades later, MED was reported for the first time in the southernmost region of the country^29^. This introduction most likely came from the border countries, Argentina and Uruguay, that had previously reported this species^30^. Finally, the more recent survey of whiteflies in Brazil done in 2015/2016, reported a second invasion of MED in the States of São Paulo and Paraná, associated to ornamental plants^31^.

Most of the surveys for *B. tabaci* species and endosymbionts identification conducted were carried out in the South and Southeast regions of Brazil^12,28,29,31–33^. Consequently, there was a lack of information about the current distribution of the whiteflies in important agricultural regions of Brazil such as the Midwest and Northeast. These regions are the main suppliers of soybean [*Glycine max* (Linnaeus) Merrill (Fabaceae)] and cotton [*Gossypium hirsutum* Linnaeus (Malvaceae)] that are very important crops for the country’s economy. In recent years, these crops have become heavily affected by *B. tabaci*. Continuous *B. tabaci* species population studies are important because they provide useful information for the development of pest management models and may indicate the distribution and abundance of *B. tabaci* over different crop systems. In Israel, it was observed that MEAM1 populations dominate crops grown in open field, while MED populations gradually dominate crops grown in protected areas^34^ and that MEAM1 is more competitive than MED under untreated conditions with low insecticide application^35^. In Fujian - China, different aspects such as a progress of protected crops and a decline in insecticide resistance in MEAM1 favored the establishment of MED^36^.

Previously, a study was carried out and revealed the bacterial endosymbiont localization and diversity from whitefly species collected in São Paulo, Bahia, Minas Gerais and Paraná States, before MED introduction^37^. Herein, we present an updated overview of the whiteflies species status as well as their bacterial endosymbionts relationships in the Brazilian territory. The sampling was composed of 237 different locations collected between the years of 2013 and 2017 in 11 different States and different hosts found in open fields, greenhouses and flower shops. Brazil is a country with an extensive area and different crop systems. The data obtained in this study is essential to understand the current distribution of this pest after MED invasion in the country. Monitoring the distribution of whiteflies can help to prevent MED from spreading across the country and will aid for a proper management of this pest. The data suggests that MEAM1 is still the predominant species of *B. tabaci* in Brazil. However, MED is emerging as a problem on ornamental plants, which seems to be spreading this species over the country. In addition, MED was found on vegetables such as sweet pepper, tomato and cucurbits in greenhouses and on cucurbits and broccoli on open field, but was not associated to virus-infected plants.

## Results

### Host plants, geographic distribution and virus detection

The data on geographical distribution of whiteflies and their host plants can be found in Supplementary Table 1: The exact site of collection of the whiteflies can be visualized on GoogleMyMaps (https://drive.google.com/open?id=13sZxldQScbxCxb-oJcVk-C2KnYU&usp=sharing). A predominance of MEAM1 species was found across all Brazilian sampling sites. Most of the whitefly samples identified as MEAM1 were found in both open fields and greenhouses of several crops such as soybeans, tomatoes, cottons, beans, potatoes [*Solanum tuberosum* Linnaeus (Solanaceae), eggplants [*Solanum melongena* Linnaeus (Solanaceae), squashes [*Cucurbita* spp. (Cucurbitaceae)], sweet peppers [*Capsicum annuum* (Linnaeus) (Solanaceae)], cabbages [*Brassica oleracea* var. capitata Linnaeus (Brassicaceae)], muskmelons [*Cucumis melo* Linnaeus (Cucurbitaceae)], ornamental plants and weeds. Except for Santa Catarina State, MEAM1 was present in the other 10 States where the collections were carried out. MEAM1 was associated with three whitefly transmitted viruses: *Cowpea mild mottle virus* (CpMMV) in soybean from São Paulo and Bahia States (sampling site 185 and 223, respectively); *Tomato chlorosis virus* (ToCV) and *Tomato severe rugose virus* (ToSRV) in tomato from São Paulo State (sampling site 136).

**Figure 1:**
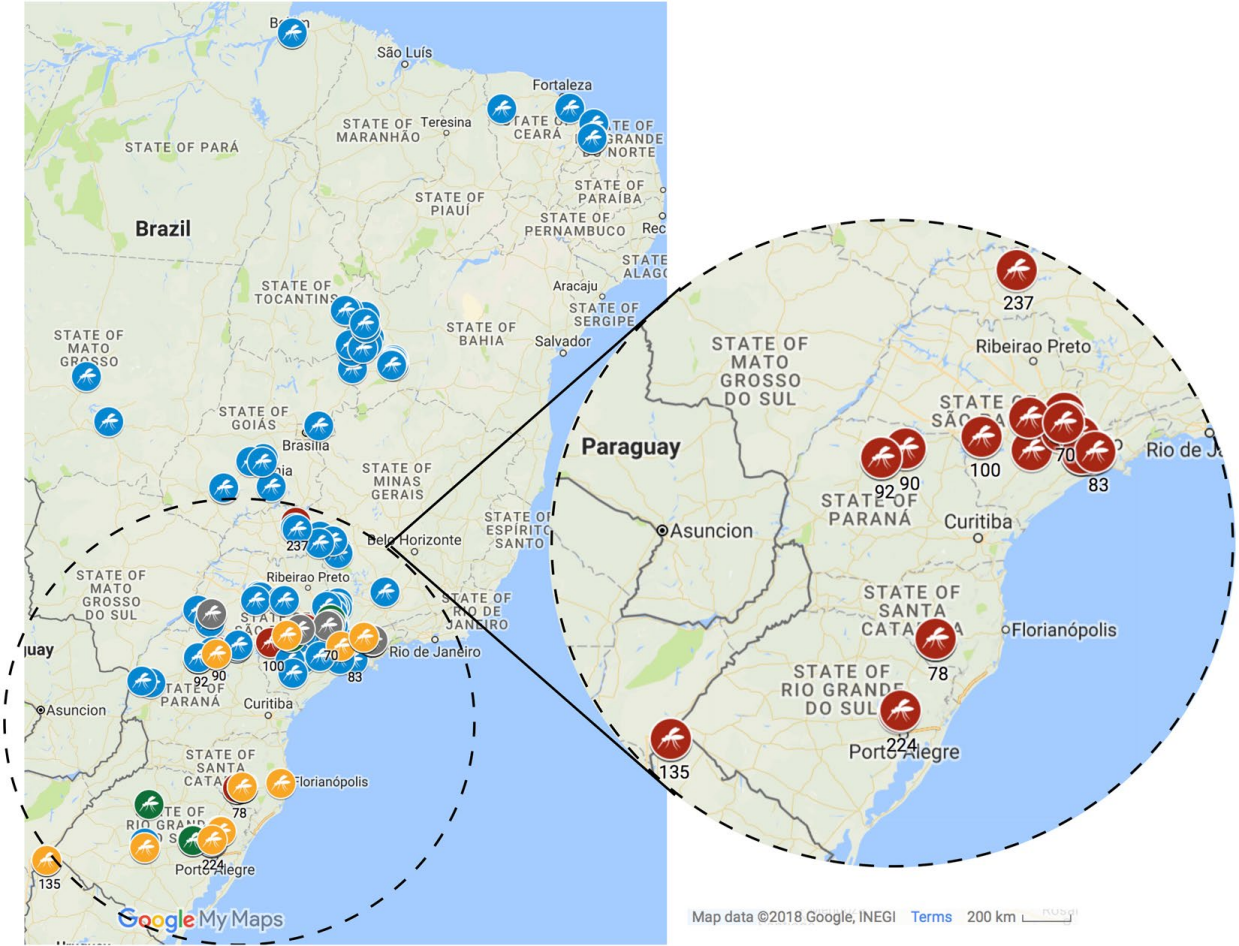
Distribution of the 237 samples of whiteflies collected in Brazil between the years of 2013 and 2017, with species colored in blue (*Bemisia tabaci* Middle East-Asia Minor 1), red (*B. tabaci* Mediterranean), green (*B. tabaci* New World), grey *Bemisia tuberculata* and yellow *Trialeurodes vaporariorum*. MED species was only present in the States of Minas Gerais, São Paulo, Paraná, Santa Catarina and Rio Grande do Sul and its distribution is highlighted. The map with all the details can be visualized on the GoogleMyMaps link (https://drive.google.com/open?id=13sZxldQScbxCxb-oJcVk-C2KnYU&usp=sharing). Map data: Google, INEGI Imagery 2017, TerraMetrics.

MED species was found in Minas Gerais, São Paulo, Paraná, Santa Catarina and Rio Grande do Sul States (Fig. 1) colonizing ornamental plants as was recently observed^31^ and on tomatoes and sweet pepper plants cultivated in greenhouses, in which ornamentals were previously grown. It was also found on cucurbits and broccoli in open fields near greenhouses where MED was previously detected. Interestingly, MED was found heavily infesting a sweet pepper (greenhouse) in the county of Cerqueira Cesar (São Paulo State) with no apparent connection and kilometers apart from any ornamental crop. The sweet pepper plants were all symptomless and no viruses were detected in ornamental plants associated to MED.

The native species of *B. tabaci*, New World (NW), were found mainly on the weeds: *Sida* spp. (Malvaceae), *Euphorbia heterophylla* Linnaeus (Euphorbiaceae), *Ageratum* spp. (Asteraceae) but also colonizing soybeans in São Paulo and Rio Grande do Sul States. The predominant species found on soybean in Rio Grande do Sul State was NW.

The greenhouse whitefly, *Trialeurodes vaporariorum* (Westwood) (Hemiptera: Aleyrodidae), was identified in the States of Rio Grande do Sul, Santa Catarina, São Paulo, Paraná and Minas Gerais mostly in greenhouses. The crinivirus, ToCV, was detected in tomatoes (greenhouse) in Rio Grande do Sul (sampling site 132), heavily infested with *T. vaporariorum*.

The predominant whitefly found in cassava [*Manihot esculenta* (Crantz) (Euphorbiaceae)] was *Bemisia tuberculata* (Bondar) (Hemiptera: Aleyrodidae).

A summary of results of virus detection can be found in Supplementary Table 2.

### Endosymbiont diversity

**Figure 2:**
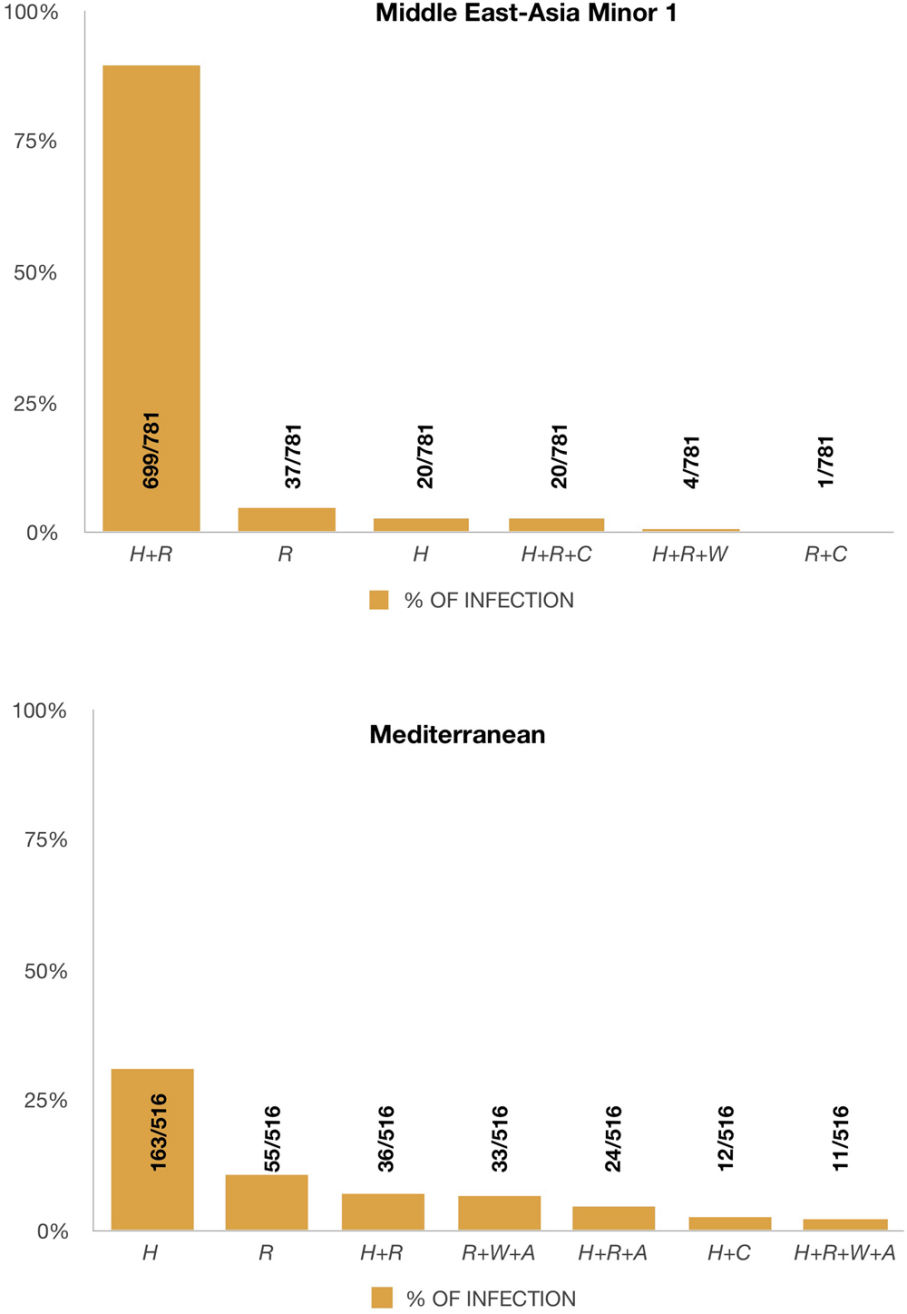
Coinfections of facultative endosymbionts found in populations of *Bemisia tabaci* Middle East-Asia Minor 1 and Mediterranean from Brazil. Numbers inside the axis represent the number of individuals harboring the set of endosymbionts/number of tested individuals. Individuals infected by a single facultative endosymbiont are represented by only one letter, individuals with co-infections are represented by the plus sign (+). H: *Hamiltonella*; R: *Rickettsia*; C: *Cardinium*; W: *Wolbachia*; A: *Arsenophonus*.

The primary endosymbiont *Portiera aleyrodidarum* was detected in all the whiteflies analyzed including *B. tuberculata* and *T. vaporariorum*. The main co-infection observed in MEAM1 populations was *Hamiltonella* and *Rickettsia*, these facultative endosymbionts were observed in 699 specimens out of 781 samples analyzed (89, 5%) (Fig. 2). In addition, *Cardinium* and *Wolbachia* were also found infecting MEAM1.

The co-infections present in MED populations were highly variable with different combinations of *Hamiltonella*, *Rickettsia*, *Arsenophonus, Cardinium* and *Wolbachia*. However, MED specimens were predominantly single-infected by *Hamiltonella* (163 specimens out of 516 samples) (Fig. 2).

New World populations were infected by different combinations of *Hamiltonella*, *Cardinium*, *Wolbachia*, *Arsenophonus* and *Fristchea*. For *T. vaporariorum* only *Arsenophonus* was identified and for *B. tuberculata*, *Rickettsia*, *Wolbachia* and *Arsenophonus* were identified (Supplementary Table 1).

**Figure 3:**
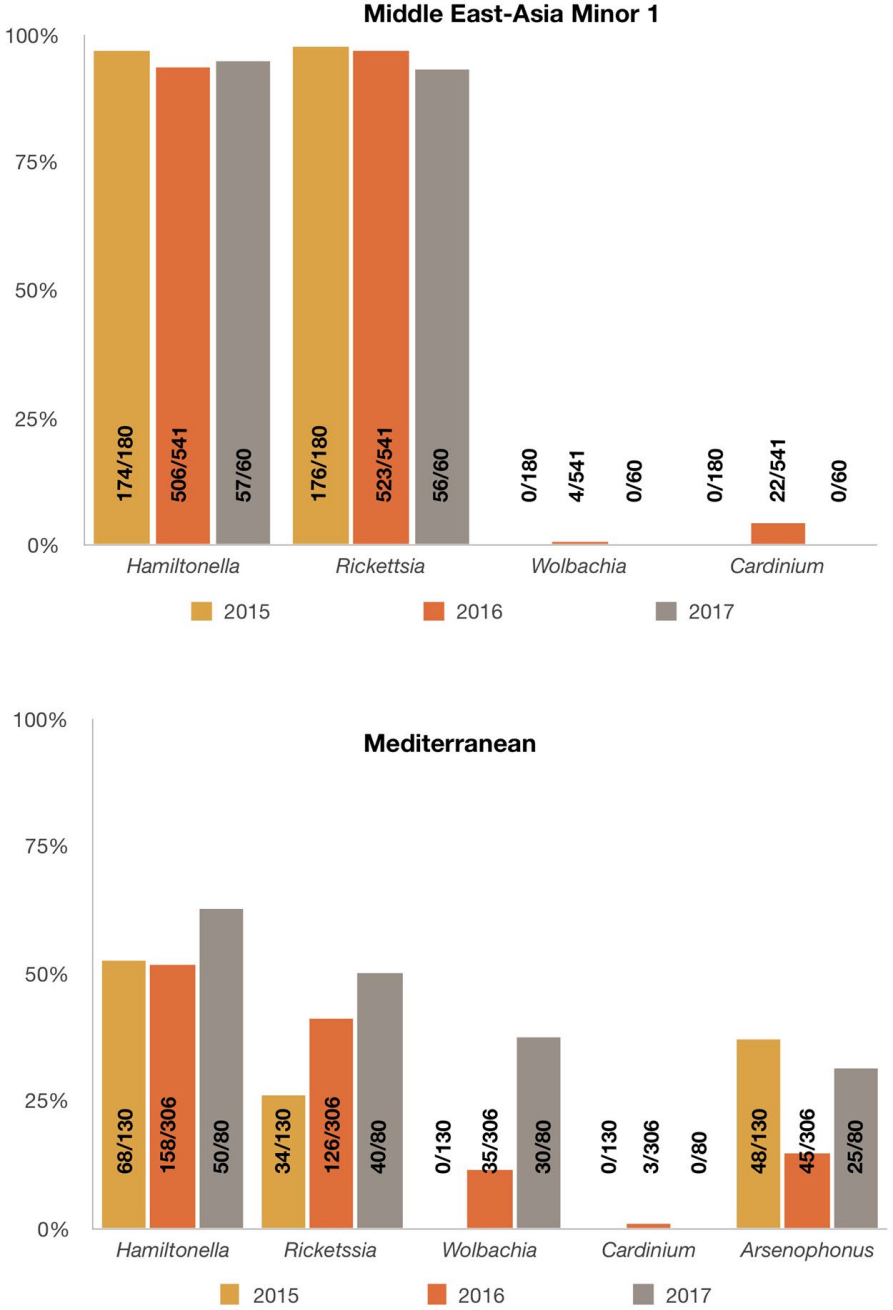
Facultative endosymbiont infection rates from *Bemisia tabaci* Middle East-Asia Minor 1 and Mediterranean populations from Brazil collected in 2013–2017. Numbers inside the axis represent the number of individuals harboring the endosymbionts/number of tested individuals.

The distribution of endosymbionts in MED was heterogeneous showing an increase in the percentage of *Hamiltonella*, *Rickettsia* and *Wolbachia* from 2015 to 2017. In contrast, the endosymbiont distribution remained homogeneous for MEAM1 species and the percentages of all the endosymbionts identified remained similar between 2015 and 2017 (Fig. 3).

In addition, it was noticed the presence of whiteflies populations that were free of facultative endosymbionts. This was reported in some MEAM1 individuals from sampling sites 21 and 35; some MED individuals from sampling sites 83, 84, 85, 89, 91, 100, 103, 106, 109, 120, 121, 128, 130, 131, 160 and 162; *T. vaporariorum* individuals from sampling sites 86, 87, 88 and 157. Curiously, most of these individuals were present in weeds and ornamentals plants. However, they were also found in crops such as potato, tomato and cucumber plants.

### Whitefly phylogenies

**Figure 4:**
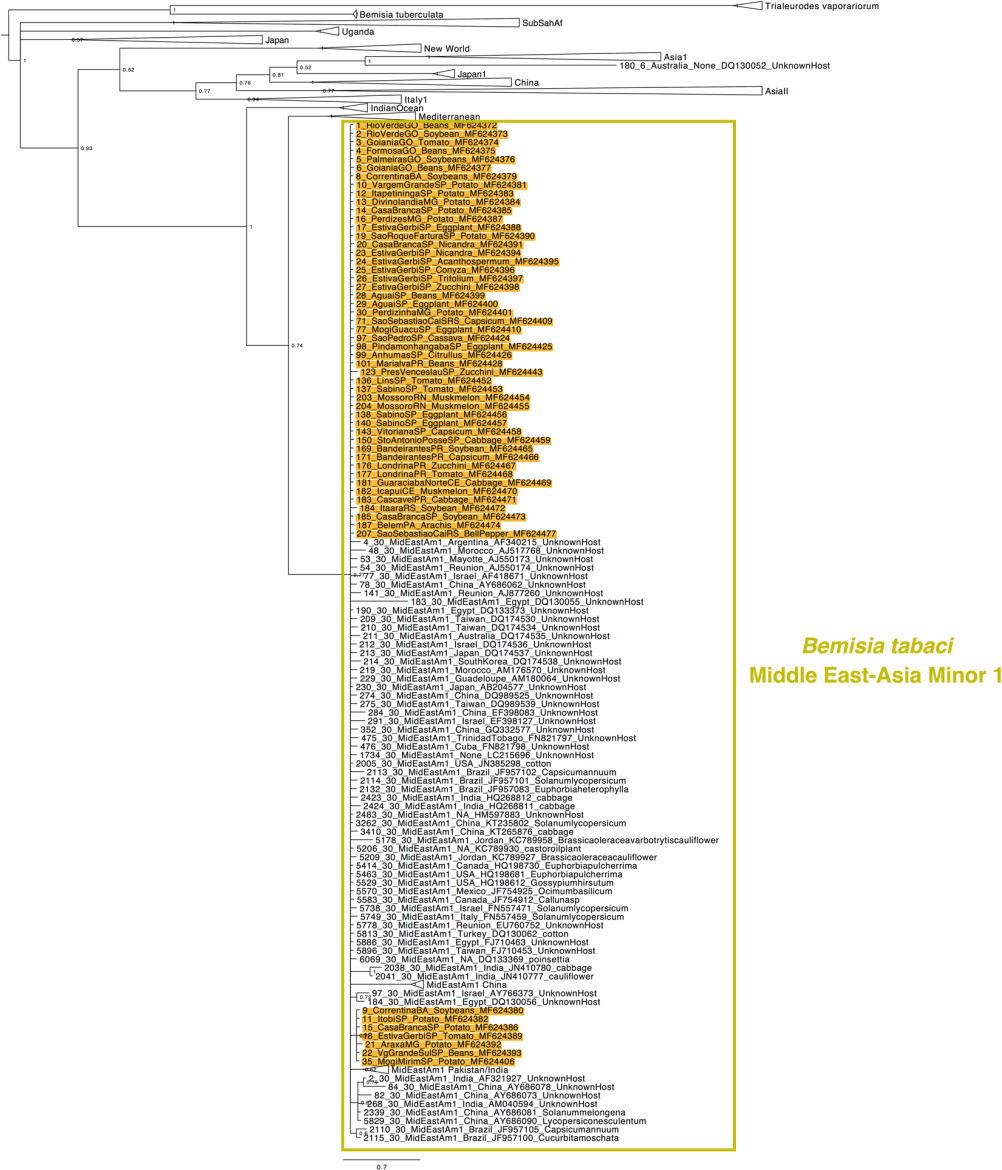
Phylogenetic tree of the partial mtCOI gene from Middle East-Asia Minor species of *Bemisia tabaci* complex including 1034 sequences conducted using MrBayes v. 3.2.2 on the Magnus supercomputer. Sequences obtained in this work are highlighted in yellow. The host and city of collection are indicated.

**Figure 5:**
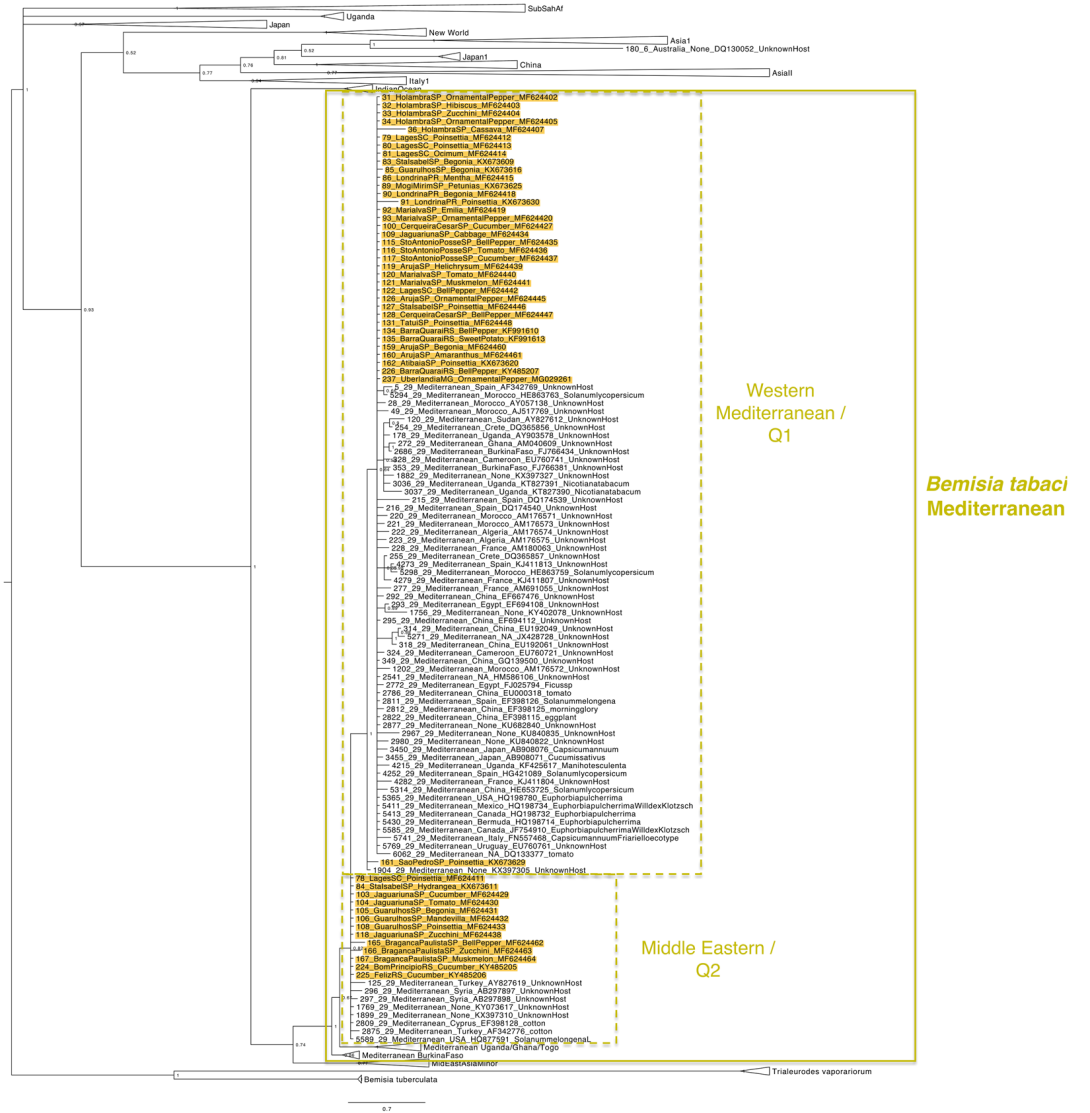
Phylogenetic tree of the partial mtCOI gene from Mediterranean species including 1034 sequences conducted using MrBayes v. 3.2.2 on the Magnus supercomputer. Sequences obtained in this work are highlighted in yellow. The host and city of collection are indicated.

**Figure 6:**
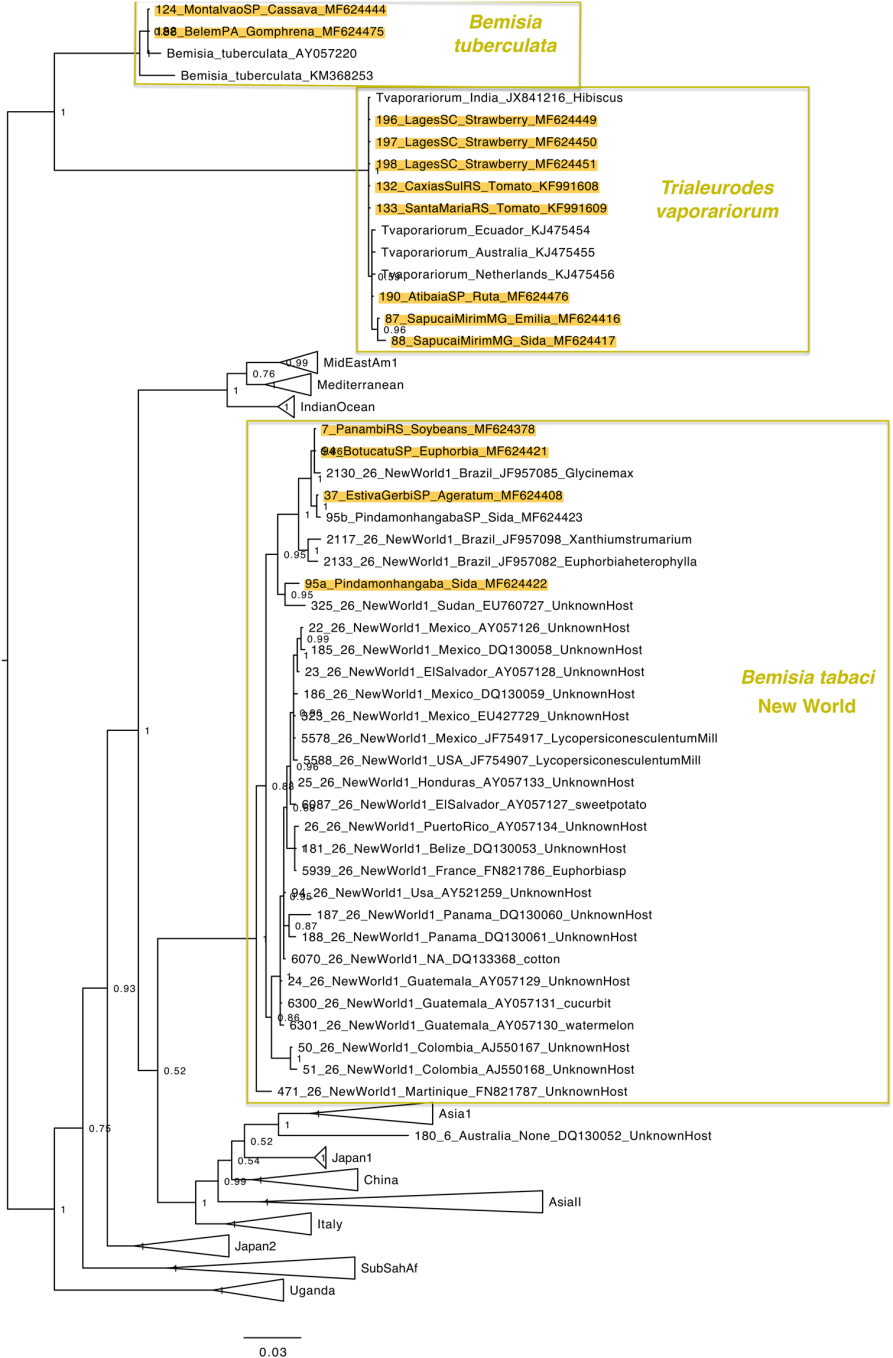
Phylogenetic tree of the partial mtCOI gene from New World, *Trialeurodes vaporariorum* and *Bemisia tuberculata* species including 1034 sequences conducted using MrBayes v. 3.2.2 on the Magnus supercomputer. Sequences obtained in this work are highlighted in yellow. The host and city of collection are indicated.

The whitefly phylogenetic tree was divided in three figures for better visualization, one showing the clades of Middle East-Asia Minor group (Fig. 4), one showing the clades of Mediterranean (Fig. 5) and the other showing the clades of New World, *T. vaporariorum* and *B. tuberculata* (Fig. 6). The phylogenetic tree revealed a genetically homogeneous population of MEAM1 across the country, regardless of the host or the production system (greenhouse or open field) (Fig. 4). The MEAM2 clade is not present in the phylogenetic tree due to a recent study suggesting that MEAM2 is a pseudogene artifact and so not a real species^38^. In contrast, MED populations in Brazil seem to be more variable genetically as two distinct genetic groups were observed, Western Mediterranean (Q1) and Middle Eastern (Q2) (Fig. 5). It seems to have no correlation between specific endosymbiont constitution and populations separated in different sub-clades observed for MED.

Populations of *T. vaporariorum* analyzed presented low diversity among them. However, samples from the South Region of the country (Santa Catarina and Rio Grande do Sul) were grouped in a different sub-clade from samples collected in the Midwest Region (São Paulo and Minas Gerais States) (Fig. 6).

### Facultative endosymbiont phylogenies

**Figure 7:**
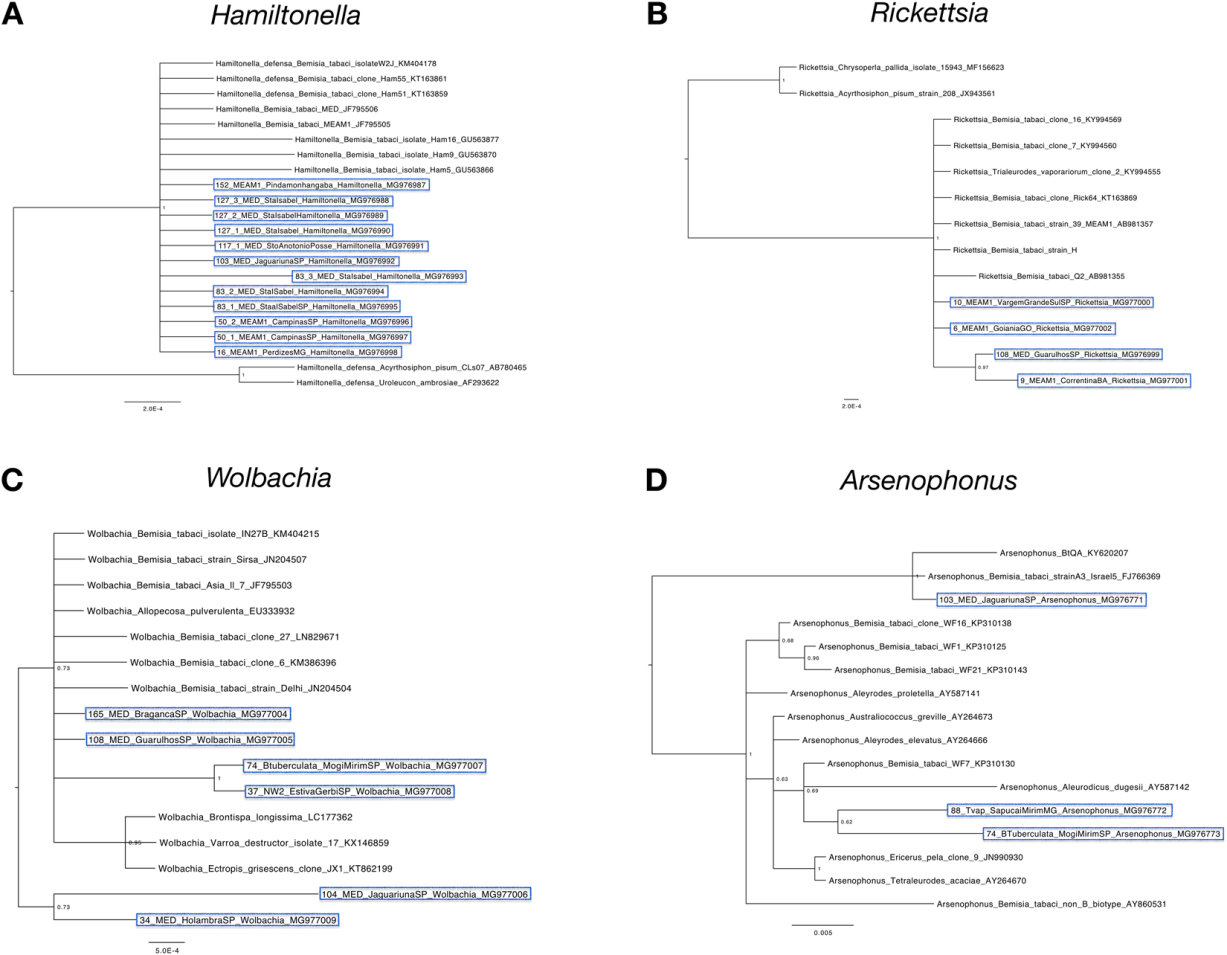
Phylogenetic tree of facultative endosymbiont *Hamiltonella* (**A**), *Rickettsia* (**B**), *Wolbachia* (**C**) and *Arsenophonus* (**D**) populations present in *Bemisia tabaci* from Brazil. Partial 16S rDNA gene was analyzed for *Hamiltonella*, *Rickettsia* and *Wolbachia* and partial 23S rDNA gene analyzed for *Arsenophonus*. Analyses were conducted using MrBayes v. 3.2.2 on the Magnus supercomputer. Sequences obtained in this work are highlighted in blue.

Phylogenetic analysis was carried for the most abundant facultative endosymbionts *Hamiltonella*, *Rickettsia*, *Wolbachia* and *Arsenophonus* (Fig. 7).

The *Hamiltonella* phylogenetic tree (Fig. 7A) as well as the *Rickettsia* (Fig. 7B) grouped different endosymbiont populations from MED and MEAM1 in the same clades, suggesting low genetic diversity of both *Hamiltonella* and *Rickettsia* among the whitefly populations analyzed.

The phylogenetic analysis of *Wolbachia* (Fig. 7C) separated the endosymbionts populations from MED collected in the same State (São Paulo) in two different main clades. In addition, *Wolbachia* populations from NW and *B. tuberculata* were grouped in a sub-clade.

Finally, phylogenetic analysis of *Arsenophonus* (Fig. 7D) grouped endosymbiotic populations found in *T. vaporariorum* and *B. tuberculata* in a different clade from *Arsenophonus* found in MED.

## Discussion

Herein, we report the current scenario of geographical distribution, phylogenies, hosts, endosymbionts and main viruses associated to whiteflies in Brazil four years after MED introduction^29^ and 27 years after MEAM1 introduction^28^. The invasive MEAM1 is still the prevalent species across the country with an established set of endosymbiont, low genetic diversity and is the main species associated to plant viruses. On the other hand, MED was not found in the Northern States and is mainly found in ornamental plants. In addition, MED presents two distinct genetic groups with a highly variable endosymbionts constitution and is not associated to plant viruses yet.

The first report of MED in Brazil has posed many questions regarding the possible impacts on the local agriculture^29^. MED is known to be able to out-compete MEAM1 under conditions of high insecticides use^36,39,40^. The displacement of MEAM1 by MED in major crops such as soybeans and cotton would change the entire pest management system and tactics of control of this pest in Brazil. MED invasion into row crops could increase the cost of production and threaten the whole agricultural system. In this study, our survey revealed this displacement has not occurred yet and MEAM1 is still likely to be the prevalent species in major crops in important agricultural regions of Brazil in the Midwest and Northeast. However, the presence of MED in Minas Gerais, São Paulo, Paraná, Santa Catarina and Rio Grande do Sul States is still threatening Brazilian agriculture and a preventive management must be carried out to avoid the spreading of MED to other regions. Although MED is still strongly associated to ornamental plants or vegetables cultivated in greenhouses, we also found this species colonizing plants in open field close to greenhouses with ornamental plants, which is a different scenario than the one verified two years before^31^. Recent studies in Florida revealed that after twelve years in which MED, was associated with greenhouse-grown ornamental horticulture, it started also to be detected in residential landscapes and open field agricultural production in 2016^41^. In Florida, different factors may explain the detection of *B. tabaci* MED in the landscape, such as changes in the use of insecticides that are less effective to MED and environmental conditions that may have been favorable for the buildup of *B. tabaci* in this scenery. MED was also recently detected in open-field bean crop in Argentina, together with MEAM1 and NW^42^. *B. tabaci* MED is not usually a problem in the landscape, but the tropical weather and presence of cultivated hosts and weeds during the year can contribute for the adaptation of MED to the landscape and dynamic of the insect in these regions needs to be carefully surveyed.

The emergence of viruses in several regions may also be related to presence of a specific species of whitefly. In Brazil, this was the case for begomoviruses in tomatoes after MEAM1 invasion^43^ and the widespread of *Bean golden mosaic virus* (BGMV) in beans^44^. Concerning the MED species, in China for example, the *Tomato yellow leaf curl virus* (TYLCV) became an emerging virus after the detection of MED species^45^ that caused the displacement of both MEAM1 and the native species in this country^46,47^. A similar situation has been reported in Mediterranean countries^48^. TYLCV, one of the most devastating viruses in the world, has not yet been reported in Brazil according to previous surveys^49^. However, it has been reported in the neighboring country, Venezuela^50^, being a menace to the Brazilian agriculture. In this study, virus infected plants were mainly associated to MEAM1 and the viruses identified were CpMMV for soybean; ToSRV and ToCV for tomatoes. However, it is known that whiteflies in Brazil are also transmitting the begomovirus (BGMV), causing several economic losses to the bean crop. BGMV was not identified in the sampling sites where this survey was carried out.

Other studies suggest the higher ability of MED to colonize pepper compared to MEAM1^51,52^ as well as the predominance of MED over MEAM1 due to the intensive use of insecticides^36^. It’s interesting to mention that we found sweet pepper plants heavily infested with MED species, under high pressure of insecticide use, in a greenhouse with no connection with ornamental plants, and it was impossible to presume the origin of MED is this region. In this study, virus symptoms were not observed on sweet pepper plants infested with MED species suggesting that these whiteflies may not be as successful in transmitting viruses as MEAM1.

The Native American *B. tabaci* species (NW) was found on weeds (*Sida* spp., *Euphorbia heterophylla* and *Ageratum* spp.) and was the prevalent species in soybean in Rio Grande do Sul, the southernmost State of Brazil. It was thought that the indigenous species from the Americas would have been completely displaced by MEAM1 in Brazil. However, they were reported since 2012 colonizing *E. heterophylla*, *Xanthium cavanillesii* Schouw (Asteraceae), soybean^12^ and *Ipomoea* sp. (Convolvulaceae)^33^. The NW species was demonstrated to be a good vector of the begomovirus, BGMV and the carlavirus, CpMMV, to beans^53^. In this study, no viruses were associated to plants where NW species were collected. In Argentina, NW was also reported in soybeans and beans^54^, which may indicate a suitable condition for this species to become a concern to the these crops in this region. In Brazil, *B. tuberculata* has been previously reported infesting cassava^37^. In this study, all whiteflies collected on cassava were identified as *B. tuberculata*, except for sample ID 36 in which *B. tabaci* MED adults were found. Even though the report of a *B. tabaci* species on cassava in Brazil is unusual, it’s not possible to prove whether MED was actually colonizing and completing the life cycle on this cassava plant since only adults were analyzed and they could be only feeding on this cassava plant. A different situation is observed in African countries where different species of *B. tabaci* heavily infest cassava, transmitting important viruses and causing severe damages to this crop there^55^. There are no reports of whitefly-transmitted viruses to cassava in Brazil to date.

In this study, *T. vaporariorum* was detected mainly in greenhouses. *T. vaporariorum* is a vector of the crinivirus *Tomato chlorosis virus*, ToCV^56^ and *Tomato infectious chlorosis virus*, TiCV^57^. The later has not yet been reported in Brazil. ToCV is also transmitted by *B. tabaci* and is becoming a serious threat for tomato production in Central Brazil^58^. In this survey, ToCV infected tomato plants were found in a greenhouse in Rio Grande do Sul State highly infested by *T. vaporariorum*, reinforcing the importance of this species as a vector.

The phylogenetic analysis of the mtCOI gene suggested very low genetic variability among MEAM1 populations. MEAM1 individuals collected in Rio Grande do Sul State (Southernmost region) and Pará State (Northernmost Region) were in the same branch in the Phylogenetic Tree (Fig. 3), indicating that the population of MEAM1 in Brazil is very homogeneous across the country and all the individuals are very close genetically. This could also be verified by testing the endosymbiont composition which was very homogeneous for MEAM1 populations as 89,5% of the specimens analyzed presented a co-infection of *Hamiltonella* and *Rickettsia* (Fig. 2). In contrast, mtCOI analysis of MED revealed a highly variable population, as individuals collected in the same State (São Paulo State) were placed in distinct genetic groups in the Phylogenetic Tree (Fig. 5). The facultative endosymbionts found in MED also revealed high variability (Fig. 2). Many different facultative endosymbionts were found infecting MED populations. However, most of the specimens analyzed (31%) were infected only by *Hamiltonella* (Fig. 2). It was possible to verify a co-relation between sampling sites and endosymbionts for MED (Supplementary Table 1). The predominant facultative endosymbionts in populations from Bragança Paulista/SP (sampling sites 165, 166 and 167) was *Rickettsia*, *Wolbachia* and *Arsenophonus*. In Holambra/SP (sampling sites 31, 32, 33, 34) the predominant endosymbionts was *Hamiltonella*, *Rickettsia*, *Arsenophonus*, and *Wolbachia*. In Lages/SC (sampling site 78, 79, 80, 81) and Marialva/SP (sampling site 120 and 121) most of the specimens were infected only by *Hamiltonella*. In Santa Isabel/SP (sampling site 83 and 84), the predominant endosymbionts were *Hamiltonella*, *Rickettsia* and *Arsenophonus*. In Guarulhos/SP (sampling site 105 and 106) most of the specimens were infected only by *Rickettsia*. In Barra do Quaraí/RS, the predominant facultative endosymbionts were *Hamiltonella* and *Cardinium*. This highly variable set of endosymbionts suggests different introductions of MED species in Brazil (also observed by^31^).

The presence of whitefly individuals free of facultative endosymbionts occurred mainly in MED populations in this survey. The absence of facultative endosymbionts in populations must be reported and may have biological implications on the whitefly vector such as better virus acquisition and retention, and a faster development time^59^.

Endosymbionts analyses of MED over the years revealed an increasing percentage of *Hamiltonella*, *Rickettsia* and *Wolbachia* from 2015 to 2017 (Fig. 3). This potentially indicates different introductions of MED species in Brazil. It is already known that secondary endosymbiont composition differs among different MED groups^60,61^. No correlation between facultative endosymbionts and different genetic groups of MED (Western Mediterranean and Middle Eastern) was observed in this study.

The high incidences of *Hamiltonella* in Brazilian populations of *B. tabaci* may have serious implications to virus transmission in agriculture. *Hamiltonella* has been identified as a key factor in the transmission of the begomovirus, *Tomato yellow leaf curl virus* (TYLCV) encoding a GroEL chaperonin homologue protein that safeguards begomoviruses in the haemolymph^62^. The effects of *Hamiltonella* in the transmission of plant viruses found in Brazil are still unknown.

The phylogenetic analyses of the endosymbionts suggest that *Hamiltonella* and *Rickettsia* from MED and MEAM1 are genetically close, which might be a result of horizontal transmission of these endosymbionts between different whitefly species. Samples from different States were placed in the same clades in the phylogenetic tree (Fig. 7A,B).

The analysis of *Wolbachia* has placed the endosymbionts present in MED species in different clades (Fig. 7C). Curiously, samples in the upper clade (165 and 108) are geographically closer from each other and kilometers apart from samples in the bottom clade (34 and 104), that are located in neighboring counties (Supplementary Figure 1). Besides, *Wolbachia* was detected only in MED, MEAM1, NW and *B. tuberculata* in São Paulo State, excepted by one specimen (NW) in Rio Grande do Sul (sampling site 7). The restriction of *Wolbachia* to São Paulo State may indicate a possible event of horizontal transmission among different species of whiteflies.

Horizontal transmission of endosymbionts between whitefly populations can be influential to the agriculture system. The acquisition of an endosymbiont that was not present in the population may rapidly change the biology of the insect. Previous studies reported that *Rickettsia*-infected whiteflies produced more offspring, had higher survival to adulthood, developed faster, and produced a higher proportion of daughters compared with uninfected whiteflies^63^.

In summary, this is the most comprehensive study on surveying and identifying whiteflies species in Brazil, which showed the distribution, the phylogenetic relationships and the coinfection of endosymbionts from the main species found in the country. This kind of survey is essential for the determination of the suitable management strategies of these pests. This study opens new doors and raises questions that must be further clarified over MED behavior in Brazil. Biological aspects must be verified, such as the insecticide resistance under the Brazilian conditions, the competition with the MEAM1 species in different plant hosts, and its ability to transmit local begomoviruses and criniviruses.

## Methods

### Whitefly sampling

Adults of whiteflies (*B. tabaci*, *B. tuberculata* and *T. vaporariorum)* were collected from different host plants including vegetables, ornamentals and weeds from open fields, greenhouses and flower shops between March 2013 and April 2017 in 11 States of Brazil. Collection was performed using a hand-held aspirator, transferring insects immediately to 90% ethanol and storing them at −20 °C until further molecular identification of the whitefly species and their endosymbionts detection. The sites and times of collection, host plants and geographical coordinates are summarized in Supplementary Table 1. Geographical coordinates of each sample were added to GoogleMyMaps and can be visualized on-line (https://drive.google.com/open?id=13sZxldQScbxCxb-oJcVk-C2KnYU&usp=sharing).

### DNA extraction and whitefly species identification

Total nucleic acids were extracted from each individual whitefly, following a modified Chelex protocol^64^. Ten insects for each population were individually tested, except for populations ID’s 3, 7, 90, 95, 125 and 179 where fewer insects were collected and analyzed. Populations with mixture of different species were identified with letters in the population ID (Supplementary Table 1). *B*. *tabaci* adults were homogenized in 60 µl of 5% Chelex solution in a 1.5 ml tube. The tube was vortexed for few seconds, and then incubated at 56 °C for 15 min and at 99 °C for 8 min. After centrifugation at 13000 rpm for 5 min, the supernatant was then collected and used as a template for the PCR amplification. Primers sequence and annealing temperatures of PCR reactions used for whiteflies identification are available on Supplementary Table 3.

All DNA samples were first subjected to PCR analysis to differentiate MEAM1 from MED using the primers pair Bem23F and Bem23R, which amplifies a microsatellite locus of about 200 bp and 400 bp for MEAM1 and MED, respectively^34,65,66^. Later, samples that did not amplify were screened with the generic insect primers C1-J-2195 and TL2-N-3014 that amplify a fragment of the mtCOI^67^ followed by Restriction fragment length polymorphism (RFLP) technique^12,68^ to identify NW. RFLP analysis of the amplicons consisted of 5 µl of each PCR (880 bp) digested with one unit of *TaqI* at 65 °C for 2 hours in a final volume of 15 µl. The restricted DNA was visualized by electrophoresis in 2% agarose gel stained with ethidium bromide. Samples that still did not amplify or had unexpected RFLP pattern were analyzed with specific primers for *T. vaporariorum*, TvapF and Wfrev^69^.

A representative number (121 out of 237) of PCR products amplified from mtCOI of the whiteflies were purified (QIAquick Gel Extraction Kit Qiagen) and sequenced (Macrogen, South Korea) in both directions using C1 -J-2195/TL2-N-3014 for *B. tabaci* and *B. tuberculata* or TvapF/Wfrev for *T. vaporariorum*. The nucleotides sequences were analyzed and compared with those present in GenBank database using BLAST tools (http://blast.ncbi.nlm.nih.gov/Blast). Nucleotide sequences from mtCOI were deposited in GenBank and accession numbers are available in Supplementary Table 1.

### Molecular detection of endosymbionts

The same DNA extracted from each individual (10 insects/population) was used for screening *Portiera aleyrodidarum*^70^, and the six secondary endosymbionts *Hamiltonella*^71^, *Rickettsia*^72^, *Wolbachia*^73^, *Arsenophonus*^74^, *Cardinium*^75^ and *Fritschea*^76^ that were reported from whiteflies, using genus-specific primers targeting the 16S or 23S rDNA genes. PCR cycling was performed as described by^37^. The endosymbiont presence confirmation was performed by sequencing the amplified sequences from representative individuals. Primers sequence and annealing temperatures of PCR reactions used for endosymbiont screening are available on Supplementary Table 3.

PCR products of the facultative endosymbionts were purified (QIAquick Gel Extraction Kit Qiagen) and sequenced (Macrogen, South Korea). The nucleotides sequences were analyzed and compared with those present in GenBank database using BLAST tools (http://blast.ncbi.nlm.nih.gov/Blast). Nucleotide sequences were deposited on GenBank and accession numbers are available in Fig. 7.

### Phylogenetic analysis

Phylogenetic analyses were carried out using the 121 *B. tabaci* mtCOI sequences obtained in this study added to the new global *B. tabaci* mtCOI dataset^13^ downloaded from GenBank on November 1, 2017 and includes 1040 sequences. The mtCOI, sequences ranged from 562 to 626 bp in length. In addition, phylogenetic analyses were carried out for the facultative endosymbionts *Hamiltonella*, *Rickettsia*, *Wolbachia* and *Arsenophonus*. Partial 16S rDNA gene was analyzed for *Hamiltonella*, *Rickettsia* and *Wolbachia*. For *Arsenophonus*, partial 23S rDNA gene was analyzed. The alignment consisted of sequences of 628 bp, 743 bp, 569 bp and 606 bp in length for *Hamiltonella*, *Rickettsia*, *Wolbachia* and *Arsenophonus* respectively. Multiple sequence alignment was prepared using MAFFT^77^ within the Geneious 9.1.5 software.

Subsequently, Bayesian analyses were conducted using Mr Bayes v. 3.2.2^78^ and were run in parallel across 384 nodes on the Magnus supercomputer (located at the Pawsey Centre, Western Australia). Analyses were run for 30 million generations with sampling every 1000 generations. Each analysis consisted of four independent runs, each utilizing four coupled Markov chains. The run convergence was monitored by finding the plateau in the likelihood scores (standard deviation of split frequencies <0.0015). The first 25% of each run was discarded as burn-in for the estimation of a majority rule consensus topology and posterior probability for each node. Trees were visualized, edited and rooted using FigTree v1.4.2.

### Whitefly-transmitted virus detection

Field plants colonized by whiteflies showing typical viral disease symptoms such as mosaic leaf pattern, crinkled leaves, yellowed leaves and plant stunting were collected for further molecular analysis. Nucleic acid extractions were carried out using the Viral RNA/DNA Mini Kit by Invitrogen. Subsequently, different PCR’s were carried out for the detection of the main viral diseases for each crop in Brazil. For soybean, the detection of *Cowpea mild mottle virus* (CpMMV)^53^ was carried out, tomato plants were analyzed for the presence of *Tomato severe rugose virus* (ToSRV)^79^ and *Tomato chlorosis virus* (ToCV)^80^. Ornamental plants were analyzed for the presence of Torradovirus^81^. Primers details and PCR conditions are available in Supplementary Table 3.

## Electronic supplementary material

Supplementry Infornation
