# Supplementary material for: Distribution and phylogenetics of whiteflies and their endosymbiont relationships after the Mediterranean species invasion in Brazil
Source: Sci Rep. 2018 Oct 1;8:14589. doi: 10.1038/s41598-018-32913-1 (PMC6167372; doi:10.1038/s41598-018-32913-1)

Distribution and phylogenetics of whiteflies and their endosymbiont relationships after the Mediterranean species invasion in Brazil

Letícia Aparecida de Moraes[1], Cristiane Muller[2], Regiane Cristina Oliveira de Freitas Bueno[1], Antônio Santos[2], Vinicius Henrique Bello[1], Bruno Rossitto De Marchi[1], Luís Fernando Maranho Watanabe[1], Julio Massaharu Marubayashi[1], Beatriz Rosa Santos[1], Valdir Atsushi Yuki[3], Hélio Minoru Takada[3], Danielle Ribeiro de Barros[4], Carolina Garcia Neves[4], Fábio Nascimento da Silva[5], Mayra Juline Gonçalves[5], Murad Ghanim[6], Laura Boykin[7], Marcelo Agenor Pavan[1], Renate Krause-Sakate[1,*].

Supplementary Table 1. Site of collection, coordinates, collection date, host plant, whitefly species and set of facultative endosymbionts of whitefly individuals collected between the years of 2013 and 2017. Middle East-Asia Minor 1 (MEAM1); Mediterranean (MED); New World (NW); Open Field (OF); Greenhouse (GH); Flower Shop (FS); *Hamiltonella* (H); *Rickettsia* (R); *Wolbachia* (W); *Cardinium* (C); *Arsenophonus* (A); *Fristchea* (F).

Population ID	Site of Collection	Coordinates	Collection Date	Host Plant	GenBank	Species	Endosymbionts					
							H	R	W	C	A	F
1	Rio Verde/GO	17° 44' 47” S 50° 51' 33” W	2016	*Phaseolus vulgaris* (OF)	MF624372	MEAM1	10/10	7/10	0/10	0/10	0/10	0/10
2	Rio Verde/GO	17° 43' 41” S 50° 53' 42” W	2016	*Glycine max* (OF)	MF624373	MEAM1	10/10	10/10	0/10	0/10	0/10	0/10
3	Goiania/GO	16° 41' 29” S 49° 26' 9” W	2016	*Solanum lycopersicum* (OF)	MF624374	MEAM1	5/5	5/5	0/5	0/5	0/5	0/5
4	Formosa/GO	15° 33' 41” S 47° 24' 54” W	2016	*Phaseolus vulgaris* (OF)	MF624375	MEAM1	7/10	10/10	0/10	0/10	0/10	0/10
5	Palmeiras de Goiás/GO	16° 48' 34” S 49° 53' 36” W	2016	*Glycine max* (OF)	MF624376	MEAM1	10/10	10/10	0/10	0/10	0/10	0/10

6	Goiania/GO	16° 49' 43" S 49° 29' 16" W	2016	*Phaseolus vulgaris* (OF)	MF624377	MEAM1	10/10	10/10	0/10	0/10	0/10	0/10
7	Panambi/RS	28° 20' 38" S 53° 36' 47" W	2016	*Glycine max* (OF)	MF624378	NW	2/2	0/2	1/2	0/2	0/2	2/2
8	Correntina/BA	13° 13' 43" S 44° 41' 36" W	2016	*Glycine max* (OF)	MF624379	MEAM1	10/10	10/10	0/10	0/10	0/10	0/10
9	Correntina/BA	13° 24' 59" S 44° 39' 45" W	2016	*Glycine max* (OF)	MF624380	MEAM1	10/10	10/10	0/10	0/10	0/10	0/10
10	Vargem Grande do Sul/SP	21° 50' 26" S 47° 00' 06" W	2016	*Solanum tuberosum* (OF)	MF624381	MEAM1	7/10	10/10	0/10	0/10	0/10	0/10
11	Itobi/SP	21° 42' 45" S 46° 59' 41" W	2016	*Solanum tuberosum* (OF)	MF624382	MEAM1	10/10	10/10	0/10	0/10	0/10	0/10
12	Itapetininga/SP	23° 35' 23" S 48° 02' 27" W	2016	*Solanum tuberosum* (OF)	MF624383	MEAM1	10/10	10/10	2/10	0/10	0/10	0/10
13	Divinolandia/MG	21° 40' 05" S 46° 42' 51" W	2016	*Solanum tuberosum* (OF)	MF624384	MEAM1	10/10	10/10	0/10	0/10	0/10	0/10
14	Casa Branca/SP	21° 51' 43" S 46° 59' 25" W	2016	*Solanum tuberosum* (OF)	MF624385	MEAM1	10/10	10/10	0/10	0/10	0/10	0/10
15	Casa Branca/SP	21° 51' 43" S 46° 59' 32" W	2016	*Solanum tuberosum* (OF)	MF624386	MEAM1	10/10	10/10	1/10	0/10	0/10	0/10
16	Perdizes/MG	19° 37' 47" S 47° 18' 20" W	2016	*Solanum tuberosum* (OF)	MF624387	MEAM1	10/10	10/10	0/10	0/10	0/10	0/10

17	Estiva Gerbi/SP	22° 15' 13'' S 46° 58' 31” W	2016	*Solanum melongena* (OF)	MF624388	MEAM1	10/10	10/10	0/10	0/10	0/10	0/10
18	Estiva Gerbi/SP	22° 15' 02'' S 46° 58' 43” W	2016	*Solanum lycopersicum* (OF)	MF624389	MEAM1	10/10	10/10	0/10	0/10	0/10	0/10
19	São Roque da Fartura/SP	21° 50' 49'' S 46° 44' 12” W	2016	*Solanum tuberosum* (OF)	MF624390	MEAM1	10/10	10/10	0/10	0/10	0/10	0/10
20	Casa Branca/SP	21° 51' 46'' S 46° 59' 25” W	2016	*Nicandra* spp. (OF)	MF624391	MEAM1	10/10	10/10	0/10	0/10	0/10	0/10
21	Araxa/MG	19° 31' 52'' S 46° 56' 13” W	2016	*Solanum tuberosum* (OF)	MF624392	MEAM1	7/10	7/10	0/10	0/10	0/10	0/10
22	Vargem Grande do Sul/SP	21° 50' 26'' S 46° 50' 10” W	2016	*Phaseolus vulgaris* (OF)	MF624393	MEAM1	8/10	10/10	0/10	0/10	0/10	0/10
23	Estiva Gerbi/SP	22° 15' 15'' S 46° 58' 32” W	2016	*Nicandra* spp. (OF)	MF624394	MEAM1	10/10	10/10	0/10	0/10	0/10	0/10
24	Estiva Gerbi/SP	22° 15' 14'' S 46° 58' 31” W	2016	*Acanthospermum* spp. (OF)	MF624395	MEAM1	10/10	10/10	0/10	0/10	0/10	0/10
25	Estiva Gerbi/SP	22° 15' 15'' S 46° 58' 34” W	2016	*Conyza* spp. (OF)	MF624396	MEAM1	10/10	10/10	0/10	0/10	0/10	0/10
26	Estiva Gerbi/SP	22° 15' 07'' S 46° 58' 42” W	2016	*Trifolium* spp. (OF)	MF624397	MEAM1	10/10	10/10	0/10	1/10	0/10	0/10
27	Estiva Gerbi/SP	22° 15' 09'' S 46° 58' 25” W	2016	*Cucurbita pepo*	MF624398	MEAM1	10/10	10/10	0/10	0/10	0/10	0/10
28	Aguaí/SP	22° 02' 13'' S 46° 54' 32” W	2016	*Phaseolus vulgaris* (OF)	MF624399	MEAM1	10/10	10/10	0/10	0/10	0/10	0/10

29	Aguaí/SP	22° 02' 13'' S<br>46° 54' 33” W	2016	*Solanum melongena* (OF)	MF624400	MEAM1	10/10	10/10	0/10	0/10	0/10	0/10
30	Perdizinha/MG	19° 38’ 51” S<br>47° 23’ 16” W	2016	*Solanum tuberosum* (OF)	MF624401	MEAM1	10/10	10/10	0/10	0/10	0/10	0/10
31	Holambra/SP	22° 37' 34'' S<br>47° 03' 04” W	2016	*Capsicum* spp. (FS)	MF624402	MED	10/10	3/10	0/10	0/10	1/10	0/10
32	Holambra/SP	22° 36' 25'' S<br>47° 02' 53” W	2016	*Hibiscus* spp. (GH)	MF624403	MED	10/10	1/10	1/10	0/10	1/10	0/10
33	Holambra/SP	22° 36' 24'' S<br>47° 02' 50” W	2016	*Cucurbita pepo* (GH)	MF624404	MED	10/10	2/10	1/10	0/10	2/10	0/10
34	Holambra/SP	22° 38' 25'' S<br>47° 03' 07” W	2016	*Capsicum* spp. (FS)	MF624405	MED	10/10	3/10	4/10	1/10	3/10	0/10
35	Mogi Mirim/SP	22° 26' 46'' S<br>47° 04' 11'' W	2016	*Solanum tuberosum* (OF)	MF624405	MEAM1	8/10	8/10	0/10	0/10	0/10	0/10
36	Holambra/SP	22° 36' 26'' S<br>47° 02' 50” W	2016	*Manihot esculenta* (GH)	MF624407	MED	9/10	0/10	0/10	2/10	0/10	0/10
37	Estiva Gerbi/SP	22° 15' 13'' S<br>46° 58' 28” W	2016	*Ageratum* spp. (OF)	MF624408	NW	10/10	0/10	9/10	0/10	0/10	0/10
38	Assis Chateaubriand/PR	24° 22' 23'' S<br>53° 33' 06” W	2016	*Glycine max* (OF)	N/A	MEAM1	-	-	-	-	-	-
39	Palotina/PR	24° 18' 54'' S<br>53° 52' 8'' W	2016	*Gossypium hirsutum* (OF)	N/A	MEAM1	-	-	-	-	-	-
40	Correntina/BA	13° 19' 19'' S<br>44° 42' 17” W	2016	*Gossypium hirsutum* (OF)	N/A	MEAM1	-	-	-	-	-	-
41	Correntina/BA	13° 17' 9'' S	2016	*Gossypium*	N/A	MEAM1	-	-	-	-	-	-

		44° 42' 39" W		*hirsutum* (OF)								
42	Correntina/BA	13° 20' 14'' S<br>44° 45' 15" W	2016	*Phaseolus vulgaris* (OF)	N/A	MEAM1	10/10	10/10	0/10	0/10	0/10	0/10
43	Correntina/BA	13° 23' 22'' S<br>44° 44' 20" W	2016	-	N/A	MEAM1	-	-	-	-	-	-
44	Correntina/BA	13° 24' 10'' S<br>44° 45' 29" W	2016	-	N/A	MEAM1	-	-	-	-	-	-
45	Mogi das Cruzes/SP	23° 35' 49'' S<br>46° 09' 19" W	2016	*Solanum lycopersicum* (OF)	N/A	MEAM1	-	-	-	-	-	-
46	Tapira/MG	19° 55' 54'' S<br>46° 42' 45" W	2016	*Solanum tuberosum* (OF)	N/A	MEAM1	-	-	-	-	-	-
47	Tapira/MG	19° 54' 51'' S<br>46° 42' 41" W	2016	*Solanum tuberosum* (OF)	N/A	MEAM1	-	-	-	-	-	-
48	Tapira/MG	19° 59' 18'' S<br>46° 42' 14" W	2016	*Solanum tuberosum* (OF)	N/A	MEAM1	-	-	-	-	-	-
49	São Paulo/SP	23° 35' 17'' S<br>46° 38' 56" W	2016	*Solanum melongena* (OF)	N/A	MEAM1	-	-	-	-	-	-
50	Campinas/SP	22° 52' 14'' S<br>47° 04' 38" W	2016	*Brassica oleracea* (GH)	N/A	MEAM1	-	-	-	-	-	-
51	Campinas do Monte Alegre/SP	23° 38' 20'' S<br>48° 27' 52" W	2016	*Solanum tuberosum* (OF)	N/A	MEAM1	-	-	-	-	-	-
52	Casa Branca/SP	21° 51' 46'' S<br>46° 59' 26" W	2016	*Solanum tuberosum* (OF)	N/A	MEAM1	-	-	-	-	-	-

53	Casa Branca/SP	21° 51' 46'' S 46° 59' 27” W	2016	*Mimosa caesalpiniifolia* (OF)	N/A	MEAM1	-	-	-	-	-	-
54	Perdizes/MG	19° 23' 24'' S 47° 21' 44” W	2016	*Solanum tuberosum*	N/A	MEAM1	-	-	-	-	-	-
55	Morrinhos/MG	17° 41' 14'' S 49° 08' 54” W	2016	*Solanum lycopersicum* (OF)	N/A	MEAM1	-	-	-	-	-	-
56	Lavras/MG	21° 18' 43'' S 44° 59' 21” W	2016	*Solanum tuberosum* (OF)	N/A	MEAM1	-	-	-	-	-	-
57	Araxa/MG	19° 33' 46'' S 46° 55' 05” W	2016	*Solanum tuberosum* (OF)	N/A	MEAM1	-	-	-	-	-	-
58	Vargem Grande do Sul/SP	21° 50' 55'' S 46° 58' 31” W	2016	*Solanum tuberosum* (OF)	N/A	MEAM1	-	-	-	-	-	-
59	Vargem Grande do Sul/SP	21° 51' 55'' S 46° 59' 36” W	2016	*Solanum tuberosum* (OF)	N/A	MEAM1	-	-	-	-	-	-
60	Capão Bonito/SP	24° 03' 11'' S 48° 21' 49” W	2016	*Solanum tuberosum* (OF)	N/A	MEAM1	-	-	-	-	-	-
61	Vargem Grande do Sul/SP	21° 48' 39'' S 46° 56' 30” W	2016	*Solanum tuberosum* (OF)	N/A	MEAM1	-	-	-	-	-	-
62	Estiva Gerbi/SP	22° 14' 56'' S 46° 58' 34” W	2016	*Chenopodium quinoa* (OF)	N/A	MEAM1	-	-	-	-	-	-
63	Estiva Gerbi/SP	22° 15' 15'' S 46° 58' 45” W	2016	*Solanum lycopersicum* (OF)	N/A	MEAM1	-	-	-	-	-	-

64	Aguai/SP	22° 02' 13'' S 46° 54' 35” W	2016	*Capsicum annuum* (OF)	N/A	MEAM1	-	-	-	-	-	-
65	Aguai/SP	22° 02' 13'' S 46° 54' 39” W	2016	*Solanum gilo* (OF)	N/A	MEAM1	-	-	-	-	-	-
66	Mogi Mirim	22° 28' 08'' S 467°56'25” W	2016	*Manihot esculenta* (OF)	N/A	*B. tuberculata*	-	-	-	-	-	-
67	São Manuel/SP	22° 46' 15'' S 48° 34' 14” W	2017	*Solanum lycopercicum* (GH)	N/A	*T. vaporariorum*	-	-	-	-	-	-
68	Uberlandia/MG	17° 07' 23'' S 48° 16' 21” W	2016	*Solanum lycopersicum* (OF)	N/A	MEAM1	10/10	10/10	0/10	0/10	0/10	0/10
69	Uberlandia/MG	19° 09' 54'' S 48° 12' 51” W	2016	*Solanum lycopersicum* (OF)	N/A	MEAM1	10/10	10/10	0/10	0/10	0/10	0/10
70	Holambra/SP	22° 38' 25'' S 47° 03' 07” W	2016	*Solanum melongena* (FS)	N/A	MED	9/10	3/10	0/10	0/10	0/10	0/10
71	São Sebastião do Caí - RS	29° 31' 33'' S 51° 18' 11'' W	2016	*Capsicum annuum* (OF)	MF624409	MEAM1	-	-	-	-	-	-
72	São Sebastião do Caí - RS	29° 31' 33'' S 51° 18' 15” W	2016	*Solanum americanum* (OF)	N/A	MEAM1	-	-	-	-	-	-
73	São Sebastião do Caí - RS	29° 31' 33'' S 51° 18' 17” W	2016	*Solanum melongena* (OF)	N/A	MEAM1	10/10	10/10	0/10	0/10	0/10	0/10
74	Mogi Mirim/SP	22° 24' 59'' S 46° 59' 19” W	2016	*Manihot esculenta* (OF)	N/A	*B. tuberculata*	0/10	10/10	10/10	0/10	10/10	0/10
75	Mogi	22° 26' 44'' S	2016	*Manihot*	N/A	*Bemisia*	0/10	9/10	10/10	0/10	10/10	0/10

	Mirim/SP	47° 04' 11'' W		*esculenta* (OF)		*tuberculata*						
76	Mogi Mirim/SP	22° 27' 5'' S 47° 04' 56" W	2016	*Manihot esculenta* (OF)	N/A	*Bemisia tuberculata*	0/10	10/10	10/10	0/10	10/10	0/10
77	Mogi Guaçu/SP	22° 17' 4'' S 46° 57' 19" W	2016	*Solanum melongena* (OF)	MF624410	MEAM1	-	-	-	-	-	-
78	Lages/SC	27° 49' 19'' S 50° 23' 26'' W	2017	*Euphorbia pulcherrima* (GH)	MF624411	MED	10/10	0/10	0/10	0/10	0/10	0/10
79	Lages/SC	27° 49' 19'' S 50° 23' 29'' W	2017	*Euphorbia pulcherrima* (GH)	MF624412	MED	10/10	0/10	0/10	0/10	0/10	0/10
80	Lages/SC	27° 49'19'' S 50° 23' 33'' W	2017	*Euphorbia pulcherrima* (GH)	MF624413	MED	10/10	0/10	0/10	0/10	0/10	0/10
81	Lages/SC	27° 49' 19'' S 50° 23' 18'' W	2017	*Ocimum basilicum* (GH)	MF624414	MED	10/10	10/10	0/10	0/10	0/10	0/10
82	Vitoriana/SP	22° 46' 4" S 48° 24' 19" W	2017	*Capsicum annuum* (GH)	N/A	MEAM1	-	-	-	-	-	-
83	Santa Isabel/SP	23° 22' 20" S 46° 10' 35" W	2015	*Begonia* spp. (GH)	KX673609 (Moraes et al. 2017)	MED	7/10	4/10	0/10	0/10	6/10	0/10
84	Santa Isabel/SP	23° 22' 20" S' 46° 10' 40" W	2015	*Hydrangea macrophylla* (GH)	KX673611 (Moraes et al. 2017)	MED	7/10	5/10	0/10	0/10	9/10	0/10
85	Guarulhos/SP	23° 25' 20" S 46° 32' 42" W	2015	*Begonia* spp. (GH)	KX673616 (Moraes et al. 2017)	MED	8/10	0/10	0/10	0/10	5/10	0/10
86	Londrina/PR	23° 24' 28" S 51° 09' 47" W	2015	*Mentha rotundifolia*	MF624415	*T. vaporarior*	0/10	0/10	0/10	0/10	2/10	0/10

				(GH)		*um*						
87	Sapucaí Mirim/MG	22° 49' 55” S 45° 46' 28” W	2016	*Emilia sonchifolia* (GH)	MF624416	*T. vaporarioru m*	0/10	0/10	0/10	0/10	7/10	0/10
88	Sapucaí Mirim/MG	22°49' 57” S 45° 46' 28” W	2016	*Sida* spp. (GH)	MF624417	*T. vaporarioru m*	0/10	0/10	0/10	0/10	7/10	0/10
89	Mogi Mirim/SP	22° 25' 47” S 47° 00' 15” W	2015	*Petunia* spp. (GH)	KX673625 (Moraes et al. 2017)	MED	1/10	0/10	0/10	0/10	0/10	0/10
90	Londrina/PR	23° 18' 30” S 51° 10' 02” W	2016	*Begonia* spp. (FS)	MF624418	MED	3/3	0/3	0/3	0/3	2/3	0/3
91	Londrina/PR	23° 18' 12” S 51° 10' 05” W	2016	*Euphorbia pulcherrima* (FS)	KX673630 (Moraes et al. 2017)	MED	9/10	0/10	0/10	0/10	5/10	0/10
92	Marialva/PR	23° 31' 07” S 51° 48' 19” W	2016	*Emilia fosbergii* (GH)	MF624419	MED	10/10	4/10	0/10	0/10	3/10	0/10
93	Marialva/PR	23° 31' 06” S 51° 48' 20” S	2016	*Capsicum* spp. (GH)	MF624420	MED	10/10	1/10	0/10	0/10	0/10	0/10
94	Botucatu/SP	22° 50' 46” S 48° 26' 06” W	2015	*Euphorbia heterophylla* (OF)	MF624421	NW	10/10	0/10	0/10	10/10	0/10	10/10
95a	Pindamonhan gaba/SP	22° 54' 07” S 45° 33' 05” W	2016	*Sida* spp. (OF)	MF624422	NW	4/6	0/6	6/6	6/6	0/6	0/6
95b	Pindamonhan gaba/SP	22° 54' 07” S 45° 33' 05” W	2016	*Sida* spp. (OF)	MF624423	NW	3/4	0/4	3/4	2/4	2/4	0/4
96	Mogi Mirim/SP	22° 23' 25” S 46° 51' 30” W	2017	*Solanum tuberosum* (OF)	N/A	MEAM1	10/10	10/10	0/10	0/10	0/10	0/10

97	São Pedro/ SP	22° 34' 08” S 48° 05' 22” W	2017	*Manihot esculenta* (OF)	MF624424	*B. tuberculata*	-	-	-	-	-	-
98	Pindamonhan gaba/SP	22° 57' 24” S 45° 27' 11” W	2016	*Solanum melongena* (OF)	MF624425	MEAM1	0/10	10/10	0/10	1/10	0/10	0/10
99	Anhumas/SP	22° 19' 42” S 51° 23' 57 W	2016	*Citrullus lanatus* (OF)	MF624426	MEAM1	10/10	10/10	0/10	0/10	0/10	0/10
100	Cerqueira Cesar/SP	23° 01' 04” s 49° 10' 22” W	2016	*Cucumis sativus* (GH)	MF624427	MED	0/10	5/10	0/10	0/10	0/10	0/10
101	Marialva/PR	23° 31' 07” S' 51° 48' 13” W	2016	*Phaseolus vulgaris* (GH)	MF624428	MEAM1	10/10	9/10	0/10	0/10	0/10	0/10
102	Mogi Mirim/SP	22° 25' 31” S 46° 55' 02” W	2016	*Solanum tuberosum* (OF)	N/A	MEAM1	-	-	-	-	-	-
103	Jaguariuna/S P	22° 40' 35” S 47° 01' 49” W	2016	*Cucumis sativus* (GH)	MF624429	MED	4/10	8/10	2/10	0/10	5/10	0/10
104	Jaguariuna/S P	22° 40' 33” S 47° 01' 53” W	2016	*Solanum lycopersicum* (GH)	MF624430	MED	2/10	10/10	6/10	0/10	5/10	0/10
105	Guarulhos/SP	23° 25' 0.5” S 46° 21' 37” W	2016	*Begonia* spp. (GH)	MF624431	MED	0/10	10/10	0/10	0/10	0/10	0/10
106	Guarulhos/SP	23° 25' 05” S 46° 31' 59” W	2016	*Mandevilla* spp. (GH)	MF624432	MED	0/10	8/10	3/10	0/10	0/10	0/10
107	Santa Isabel/SP	23° 22' 32” S' 46° 10' 28” W	2016	*Begonia* spp. (GH)	N/A	MED	0/10	10/10	0/10	0/10	9/10	0/10
108	Guarulhos/SP	23° 25’ 10” S 46° 31’ 52” W	2016	*Euphorbia pulcherrima* (GH)	MF624433	MED	0/10	10/10	7/10	0/10	0/10	0/10

109	Jaguariuna/SP	22° 40' 41" S 47° 01' 46" W	2016	*Brassica oleracea* (OF)	MF624434	MED	4/10	5/10	3/10	0/10	5/10	0/10
110	Caxias do Sul/RS	29° 11′ 41" S 51° 01′ 02" W	2013	*Cucurbita* sp. (OF)	N/A	*T. vaporariorum*	-	-	-	-	-	-
111	Caxias do Sul/RS	29° 11′ 41" S 51° 01′ 04" W	2013	*Solanum lycopersicum* (GH)	N/A	*T. vaporariorum*	-	-	-	-	-	-
112	Caxias do Sul/RS	29° 11′ 41" S 51° 01′ 05" W	2013	*Fragaria ananassa* (GH)	N/A	*T. vaporariorum*	-	-	-	-	-	-
113	Caxias do Sul/RS	29° 11′ 41" S 51° 01′ 06" W	2013	*Galinsoga parviflora* (GH)	N/A	*T. vaporariorum*	-	-	-	-	-	-
114	Caxias do Sul/RS	29°12′ 55" S′ 50° 59′ 04" W	2013	*Cucurbita* sp. (GH)	N/A	*T. vaporariorum*	-	-	-	-	-	-
115	Santo Antônio de Posse/SP	22° 39' 45" S 46° 57' 23" W	2016	*Capsicum annuum* (GH)	MF624435	MED	0/10	8/10	0/10	0/10	0/10	0/10
116	Santo Antônio de Posse/SP	22° 39' 28" S 46° 57' 20" W	2016	*Solanum lycopersicum* (GH)	MF624436	MED	10/10	0/10	0/10	0/10	0/10	0/10
117	Santo Antônio de Posse/SP	22° 39' 45" S 46° 56' 12" W	2016	*Cucumis sativus* (GH)	MF624437	MED	5/10	9/10	2/10	0/10	0/10	0/10
118	Jaguariuna/SP	22° 39' 36" S 46° 57' 16" W	2016	*Cucubita pepo* (OF)	MF624438	MED	5/10	8/10	2/10	0/10	3/10	0/10
119	Arujá/SP	23° 23' 48" S 46° 22' 26" W	2016	*Helichrysum bracteatum* (GH)	MF624439	MED	4/10	1/10	2/10	0/10	0/10	0/10
120	Marialva/SP	23° 31' 10" S	2016	*Solanum*	MF624440	MED	8/10	0/10	0/10	0/10	0/10	0/10

		51° 48' 16” W		*lycopercicum* (GH)								
121	Marialva/SP	23° 31' 17” S 51° 48' 15” W	2016	*Cucumis melo* (GH)	MF624441	MED	8/10	0/10	0/10	0/10	0/10	0/10
122	Lages/SC	27° 49' 17” S 50° 23' 30” W	2017	*Capsicum* spp. (GH)	MF624442	MED	9/10	0/10	0/10	0/10	0/10	0/10
123	Presidente Venceslau/SP	21° 54' 56” S 51°49' 57” W	2016	*Cucubita pepo* (OF)	MF624443	MEAM1	10/10	10/10	0/10	0/10	0/10	0/10
124	Montalvao/SP	22° 02’ 23” S 51°19' 53” W	2016	*Manihot esculenta* (OF)	MF624444	*B. tuberculata*	0/10	0/10	10/10	0/10	10/10	0/10
125	Arujá/SP	23° 23’ 47” S 46° 22’ 22” W	2016	*Solanum lycopersicum* (GH)	N/A	MED	2/3	0/3	0/3	0/3	0/3	0/3
126	Arujá/SP	23° 23’ 5” S 46°22” 26” W	2016	*Capsicum* spp. (GH)	MF624445	MED	3/10	3/10	0/10	0/10	0/10	0/10
127	Santa Isabel/SP	23° 22' 22” S 46° 10' 35” W	2016	*Euphorbia pulcherrima* (GH)	MF624446	MED	4/10	3/10	0/10	0/10	1/10	0/10
128	Cerqueira Cesar/SP	23° 01' 04” S 49° 10' 22” W	2016	*Capsicum annuum* (GH)	MF624447	MED	0/10	9/10	0/10	0/10	0/10	0/10
129	Pindamonhan gaba/SP	22° 57' 21” S 45° 27' 12” W	2016	*Brassica oleracea* (OF)	N/A	MEAM1	10/10	10/10	0/10	0/10	0/10	0/10
130	Campinas/SP	22° 54' 20” S 47° 03' 39” W	2016	*Euphorbia pulcherrima* (FS)	N/A	MED	1/10	2/10	1/10	0/10	0/10	0/10
131	Tatui/SP	23° 20' 59” S 47° 50' 56” W	2016	*Euphorbia pulcherrima* (FS)	MF624448	*MED*	0/10	0/10	0/10	0/10	0/10	0/10
132	Caxias do	29° 12′ 55” S	2013	*Solanum*	KF991608	*T.*	-	-	-	-	-	-

	Sul/RS	50° 59′ 03” W		*lycopercicum* (GH)	(Barbosa et al. 2015)	*vaporarioru m*						
133	Santa Maria/RS	29° 43′ 15” S 53° 46′ 25” W	2013	*Solanum lycopercicum* (GH)	KF991609 (Barbosa et al. 2015)	*T. vaporarioru m*	-	-	-	-	-	-
134	Barra do Quarai/RS	30° 07′ 14” S 57° 20′ 42” W	2013	*Capsicum annuum* (GH)	KF991610 (Barbosa et al. 2015)	MED	6/10	0/10	0/10	10/10	0/10	0/10
135	Barra do Quarai/RS	30° 07’ 14” S 57° 20′ 41” W	2013	*Ipomoea batatas* (OF)	KF991613 (Barbosa et al. 2015)	MED	6/10	0/10	0/10	10/10	0/10	0/10
136	Lins/SP	21° 35' 39” S 49°43' 45” W	2015	*Solanum lycopercicum* (OF)	MF624452	MEAM1	10/10	9/10	0/10	0/10	0/10	0/10
137	Sabino/SP	21° 27' 38” S 49° 35' 25” W	2015	*Solanum lycopercicum* (OF)	MF624453	MEAM1	6/10	10/10	0/10	0/10	0/10	0/10
138	Sabino/SP	21° 27' 38” S 49° 35' 23” W	2015	*Solanum melongena* (OF)	MF624456	MEAM1	8/10	10/10	0/10	0/10	0/10	0/10
139	Sabino/SP	21° 26' 21” S 49° 34' 46” W	2015	*Cucurbita pepo* (OF)	N/A	MEAM1	10/10	10/10	0/10	0/10	0/10	0/10
140	Sabino/SP	21° 26' 09” S 49° 34' 53” W	2015	*Solanum melongena* (OF)	MF624457	MEAM1	10/10	10/10	0/10	0/10	0/10	0/10
141	Sabino/SP	21° 28' 51” S 49° 32' 17” W	2015	*Solanum melongena* (OF)	N/A	MEAM1	10/10	10/10	0/10	0/10	0/10	0/10

142	Vitoriana/SP	22° 46' 43” S 48° 24' 18” W	2015	*Solanum lycopercicum* (GH)	N/A	MEAM1	10/10	10/10	0/10	0/10	0/10	0/10
143	Vitoriana/SP	22° 46' 42” S 48° 24' 19” W	2015	*Capsicum annum* (GH)	MF624458	MEAM1	10/10	10/10	0/10	0/10	0/10	0/10
144	Itapolis/SP	21° 35' 30” S 48° 39' 52” W	2016	*Solanum melongena* (OF)	N/A	MEAM1	10/10	10/10	0/10	0/10	0/10	0/10
145	Itapolis/SP	21° 35' 56” S 48° 45' 08” W	2016	*Cucumis sativus* (GH)	N/A	MEAM1	10/10	10/10	0/10	0/10	0/10	0/10
146	Sorocaba/SP	23° 29' 02” S 47° 22' 35” W	2016	*Solanum lycopercicum* (GH)	N/A	MEAM1	10/10	10/10	0/10	0/10	0/10	0/10
147	Anhumas/SP	22° 19' 42” S 51° 23' 58” W	2016	*Cucurbita pepo* (OF)	N/A	MEAM1	10/10	10/10	0/10	0/10	0/10	0/10
148	Montalvao/SP	22° 02' 23” S 51° 19' 52” W	2016	*Cucurbita pepo* (OF)	N/A	MEAM1	10/10	9/10	0/10	0/10	0/10	0/10
149	Santo Antônio de Posse/SP	22° 37’ 12” S 46° 55' 18” W	2016	*Solanum lycopercicum* (GH)	N/A	MEAM1	9/10	9/10	1/10	0/10	0/10	0/10
150	Santo Antônio de Posse/SP	22° 39' 11” S 46° 56' 04” W	2016	*Brassica oleracea* (GH)	MF624459	MEAM1	10/10	10/10	0/10	0/10	0/10	0/10
151	Pindamonhan gaba/SP	22° 57' 14” S 45° 27' 10” W	2016	*Cucumis sativus* (OF)	N/A	MEAM1	10/10	10/10	0/10	10/10	0/10	0/10
152	Pindamonhan gaba/SP	22° 57' 13” S 45° 27' 10” W	2016	*Solanum lycopercicum* (OF)	N/A	MEAM1	10/10	10/10	0/10	0/10	0/10	0/10
153	Pindamonhan gaba/SP	22° 57' 22” S 45° 27' 13” W	2016	*Capsicum annuum* (OF)	N/A	MEAM1	10/10	10/10	0/10	8/10	0/10	0/10

154	Pindamonhangaba/SP	22° 57' 24” S 45° 27' 13” W	2016	*Solanum lycopercicum* (OF)	N/A	MEAM1	9/10	10/10	0/10	2/10	0/10	0/10
155	Pindamonhangaba/SP	22° 57' 24” S 45° 27' 11” W	2016	*Solanum melongena* (OF)	N/A	MEAM1	10/10	10/10	0/10	0/10	0/10	0/10
156	Pindamonhangaba/SP	22° 57' 25” S 42° 27' 13” W	2016	*Solanum lycopercicum* (OF)	N/A	MEAM1	0/10	10/10	0/10	0/10	0/10	0/10
157	Sapucaí Mirim/MG	22°49' 57” S 45° 46' 30” W	2016	Weed (GH)	N/A	*T. vaporariorum*	0/10	0/10	0/10	0/10	9/10	0/10
158	Sapucaí Mirim/MG	22° 49' 57” S 45° 46' 30” W	2016	*Hydrangea macrophylla* (GH)	N/A	*T. vaporariorum*	0/10	0/10	0/10	0/10	10/10	0/10
159	Arujá/SP	23° 24' 55” S 46° 17' 51” W	2016	*Begonia* spp. (GH)	MF624460	MED	10/10	0/10	0/10	0/10	0/10	0/10
160	Arujá/SP	23° 24' 46” S 46° 17' 75” W	2016	*Amaranthus* spp. (GH)	MF624461	MED	6/10	0/10	1/10	0/10	0/10	0/10
161	São Pedro/SP	22°33'03'' S 47°55’25'' W	2015	*Euphorbia pulcherrima* (FS)	KX673629 (Moraes et al. 2017)	MED	10/10	9/10	0/10	0/10	0/10	0/10
162	Atibaia/SP	23° 02' 23” S 46° 35' 01” W	2015	*Euphorbia pulcherrima* (GH)	KX673620 (Moraes et al. 2017)	MED	1/10	1/10	0/10	0/10	0/10	0/10
163	Pindamonhangaba/SP	22° 56' 05” S 45° 26' 25” W	2016	*Manihot esculenta* (OF)	N/A	*B. tuberculata*	0/10	1/10	10/10	0/10	1/10	9/10
164	Bragança Paulista/SP	23° 00' 48'' S 46° 35' 18'' W	2017	*Solanum lycopercicum* (OF)	N/A	MEAM1	9/10	10/10	0/10	0/10	0/10	0/10

165	Bragança Paulista/SP	23° 00' 54" S 46° 35' 31" W	2017	*Capsicum annuum* (GH)	MF624462	MED	0/10	10/10	10/10	0/10	8/10	0/10
166	Bragança Paulista/SP	23° 0' 57" S 46° 35' 33" W	2017	*Cucurbita pepo* (GH)	MF624463	MED	1/10	10/10	10/10	0/10	8/10	0/10
167	Bragança Paulista/SP	23° 0’ 55" S 46° 35' 35" W	2017	*Cucumis melo* (GH)	MF624464	MED	0/10	10/10	10/10	0/10	9/10	0/10
168	Bandeirantes/ PR	23° 07' 47" S 50° 23' 8" W	2015	*Commelina* spp. (GH)	N/A	MEAM1	-	-	-	-	-	-
169	Bandeirantes/ PR	23° 07' 48" S 50° 23' 11" W	2015	*Glycine max* (OF)	MF624465	MEAM1	10/10	10/10	0/10	0/10	0/10	0/10
170	Bandeirantes/ PR	23° 07' 47" S 50° 23' 8" W	2015	*Solanum lycopercicum* (GH)	N/A	MEAM1	10/10	10/10	0/10	0/10	0/10	0/10
171	Bandeirantes/ PR	23° 09' 22" S 50° 24' 49" W	2015	*Capsicum annuum* (GH)	MF624466	MEAM1	10/10	10/10	0/10	0/10	0/10	0/10
172	Bandeirantes/ PR	23° 09' 22" S 50° 24' 48" W	2015	*Brassica rapa* (GH)	N/A	MEAM1	10/10	10/10	0/10	0/10	0/10	0/10
173	Bandeirantes/ PR	23° 09' 22" S 50° 24' 49" W	2015	*Capsicum annuum* (GH)	N/A	MEAM1	10/10	10/10	0/10	0/10	0/10	0/10
174	Bandeirantes/ PR	23° 06' 35" S 50° 21' 31" W	2015	*Glycine max* (OF)	N/A	MEAM1	10/10	10/10	0/10	0/10	0/10	0/10
175	Bandeirantes/ PR	23° 06' 31" S 50° 21' 37" W	2015	*Brassica oleracea* (OF)	N/A	MEAM1	10/10	10/10	0/10	0/10	0/10	0/10
176	Londrina/PR	23° 24' 28" S 51° 09' 35" W	2015	*Cucurbita pepo* (OF)	MF624467	MEAM1	10/10	10/10	0/10	0/10	0/10	0/10
177	Londrina/PR	23° 24' 27" S 51° 09' 45" W	2015	*Solanum lycopercicum* (GH)	MF624468	MEAM1	10/10	7/10	0/10	0/10	0/10	0/10

178	Londrina/PR	23° 24' 29" S<br>51° 09' 45" W	2015	*Brassica oleracea* (OF)	N/A	MEAM1	10/10	10/10	0/10	0/10	0/10	0/10
179	Cambe/PR	23° 19' 6" S<br>51° 16' 55" W	2016	*Hydrangea macrophylla* (OF)	N/A	MEAM1	6/6	0/6	0/6	0/6	0/6	0/6
180	Marialva/PR	23° 31' 08" S<br>51° 48' 09" W	2016	*Solanum melongena* (OF)	N/A	MEAM1	10/10	9/10	0/10	0/10	0/10	0/10
181	Guaraciaba do Norte/CE	04° 09' 53" S<br>40°44' 44" W	2017	*Brassica oleracea* (OF)	MF624469	MEAM1	8/10	10/10	0/10	0/10	0/10	0/10
182	Icapui/CE	04°42' 46" S<br>37°21' 52" W	2017	*Cucumis melo* (OF)	MF624470	MEAM1	10/10	10/10	0/10	0/10	0/10	0/10
183	Cascavel/CE	04° 09' 01" S<br>38°15' 24" W	2017	*Brassica oleracea* (OF)	MF624471	MEAM1	10/10	9/10	0/10	0/10	0/10	0/10
184	Itaara/RS	29° 34' 48" S<br>53° 46' 21" W	2016	*Glycine max* (OF)	MF624472	MEAM1	10/10	10/10	0/10	0/10	0/10	0/10
185	Casa Branca/SP	21° 47' 27" S<br>47° 07' 29" W	2017	*Glycine max* (OF)	MF624473	MEAM1	10/10	7/10	0/10	0/10	0/10	0/10
186	Belém/PA	01° 24' 25" S<br>48° 22' 23" W	2016	*Brassica oleracea* (GH)	N/A	MEAM1	-	-	-	-	-	-
187	Belém/PA	01° 24' 20" S<br>48° 22' 23" W	2016	*Arachis pintoi* (GH)	MF624474	MEAM1	-	-	-	-	-	-
188	Belém/PA	01° 24' 17" S<br>48° 22' 25" W	2016	*Gomphrena* spp. (GH)	MF624475	MEAM1	-	-	-	-	-	-
189	Nova Mutum/MT	13°49' 22" S<br>55° 54' 07" W	2016	*Gossypium hirsutum* (OF)	N/A	MEAM1	-	-	-	-	-	-
190	Atibaia/SP	23° 08' 56" S<br>46° 36' 50" W	2015	*Ruta graveolens* (GH)	MF624476	*T. vaporarioru m*	-	-	-	-	-	-

191	Atibaia/SP	23° 01' 39” S 46° 35' 18” W	2015	*Conyza* spp. (GH)	N/A	MEAM1	-	-	-	-	-	-
192	Lages/SC	27° 46' 30" S 50° 12' 37" W	2017	*Solanum lycopercicum* (GH)	N/A	*T. vaporarioru m*	-	-	-	-	-	-
193	Lages/SC	27° 46' 30" S 50° 12' 37" W	2017	*Solanum lycopercicum* (GH)	N/A	*T. vaporarioru m*	-	-	-	-	-	-
194	Lages/SC	27° 46 '30" S 50° 12 '37" W	2017	*Solanum lycopercicum* (GH)	N/A	*T. vaporarioru m*	-	-	-	-	-	-
195	Lages/SC	27° 46' 30” S 50° 12' 37” W	2017	*Solanum lycopercicum* (GH)	N/A	*T. vaporarioru m*	-	-	-	-	-	-
196	Lages/SC	27° 46' 30” S 50° 12' 37” W	2017	*Fragaria × ananassa* (GH)	MF624449	*T. vaporarioru m*	-	-	-	-	-	-
197	Lages/SC	27° 46' 30” S 50° 12' 37” W	2017	*Fragaria × ananassa* (GH)	MF624450	*T. vaporarioru m*	-	-	-	-	-	-
198	Lages/SC	27° 46' 30” S 50° 12' 37” W	2017	*Fragaria × ananassa* (GH)	MF624451	*T. vaporarioru m*	-	-	-	-	-	-
199	Lages/SC	27° 46' 30” S 50° 12' 37” W	2017	*Nicotiana tabacum* TNN (GH)	N/A	*T. vaporarioru m*	-	-	-	-	-	-
200	Lages/SC	27° 46' 30” S 50° 12' 37” W	2017	*Physalis peruviana* (GH)	N/A	*T. vaporarioru m*	-	-	-	-	-	-
201	Santa Maria/RS	29° 43′ 16″ S 53° 46′ 27″ W	2013	Weed (OF)	N/A	*T. vaporarioru*	-	-	-	-	-	-

						m						
202	Barra do Quarai/RS	30° 07′ 15″ S 37° 25′ 42″ W	2013	*Solanum lycopercicum* (GH)	N/A	*T. vaporariorum*	-	-	-	-	-	-
203	Mossoró/RN	5° 14′ 24″ S 57° 20′ 41″ W	2017	*Cucumis melo* (OF)	MF624454	MEAM1	-	-	-	-	-	-
204	Mossoró/RN	5° 14′ 42″ S 37° 25′ 4″ W	2017	*Cucumis melo* (OF)	MF624455	MEAM1	-	-	-	-	-	-
205	Mossoró/RN	5° 15′ 11″ S 37° 25′ 14″ W	2017	*Cucumis melo* (OF)	N/A	MEAM1	-	-	-	-	-	-
206	Mossoró/RN	5° 14′ 52″ S 37° 26′ 3″ W	2017	*Cucumis melo* (OF)	N/A	MEAM1	-	-	-	-	-	-
207	São Sebastião do Caí/RS	29° 31' 33'' S 51° 18' 13” W	2016	*Capsicum annuum*	MF624477	MEAM1	-	-	-	-	-	-
208	Campo Verde/MT	15° 24' 59'' S 55° 4' 52'' W	2017	*Glycine max* (OF)	N/A	MEAM1	-	-	-	-	-	-
209	Santo Amaro da Imperatriz/SC	27° 39' 33'' S 48° 47' 49” W	2017	*Cucumis sativum(OF)*	N/A	*T. vaporariorum*	-	-	-	-	-	-
210	Correntina/BA	13° 32’ 16’’ S 46° 11’ 40” W	2017	*Glycine max* (OF)	N/A	MEAM1	-	-	-	-	-	-
211	São Desidério /BA	12° 30’ 00’’ S 46° 01’ 58” W	2017	*Gossypium hirsutum* (OF)	N/A	MEAM1	-	-	-	-	-	-
212	São Desidério /BA	12° 49’ 39’’ S 46° 07’ 14” W	2017	*Gossypium hirsutum* (OF)	N/A	MEAM1	-	-	-	-	-	-

213	São Desidério /BA	12° 43’ 38” S 46° 10’ 31” W	2017	*Glycine max* (OF)	N/A	MEAM1	-	-	-	-	-	-
214	São Desidério /BA	12° 54’ 33’’ S 46° 11’ 52” W	2017	*Glycine max* (OF)	N/A	MEAM1	-	-	-	-	-	-
215	São Desidério /BA	12° 40’ 55’’ S 46° 15’ 48” W	2017	*Phaseolus vulgaris* (OF)	N/A	MEAM1	-	-	-	-	-	-
216	Riachão das Neves/BA	11° 40’ 21’’ S 45° 43’ 15” W	2017	*Gossypium hirsutum* (OF)	N/A	MEAM1	-	-	-	-	-	-
217	Riachão das Neves/BA	12° 25’ 54’’ S 45° 34’ 34” W	2017	*Gossypium hirsutum* (OF)	N/A	MEAM1	-	-	-	-	-	-
218	Riachão das Neves/BA	12° 39’ 13’’ S 45° 38’ 14” W	2017	*Glycine max* (OF)	N/A	MEAM1	-	-	-	-	-	-
219	Riachão das Neves/BA	12° 48’ 19’’ S 45° 48’ 49” W	2017	*Glycine max* (OF)	N/A	MEAM1	-	-	-	-	-	-
220	Formosa do Rio Preto/BA	11° 28’ 12’’ S 46° 19’ 48” W	2017	*Glycine max* (OF)	N/A	MEAM1	-	-	-	-	-	-
221	Formosa do Rio Preto/BA	11° 27’ 41’’ S 46° 26’ 30” W	2017	*Glycine max* (OF)	N/A	MEAM1	-	-	-	-	-	-
222	Luís Eduardo Magalhães/BA	12° 05’ 11’’ S 45° 42’ 32” W	2017	*Gossypium hirsutum* (OF)	N/A	MEAM1	-	-	-	-	-	-
223	Luís Eduardo Magalhães/BA	11° 56’ 12’’ S 45° 44’ 22” W	2017	*Glycine max* (OF)	N/A	MEAM1	-	-	-	-	-	-
224	Bom Principio/RS	29°30’49’’S 51°20’38”W	2016	*Cucumis sativus* (GH)	KY485205	MED	-	-	-	-	-	-

225	Feliz/RS	29°29’03’’S 51°18’23’’W	2016	*Cucumis sativus* (GH)	KY485206	MED	-	-	-	-	-	-
226	Barra do Quarai/RS	30°07’13’’S 57°20’40’’W	2016	*Capsicum annuum* (GH)	KY485207	MED	-	-	-	-	-	-
227	Feliz/RS	29°29’03’’S 51°18’23’’W	2016	*Solanum lycopersicum* (GH)	N/A	MEAM1	-	-	-	-	-	-
228	Feliz/RS	29°29’03’’S 51°17’33’’W	2016	*Cucumis sativus* (GH)	N/A	MEAM1	-	-	-	-	-	-
229	Feliz/RS	29°29’38’’S 51°19’17’’W	2016	*Solanum lycopersicum* (GH)	N/A	MEAM1	-	-	-	-	-	-
230	Lajeado/RS	29°27’29’’S 52°00’59”W	2016	*Sonchus oleraceus* (GH)	N/A	NW	-	-	-	-	-	-
231	Cruzeiro do Sul/RS	29°30’53”S52 °01’08”W	2016	*Glycine max* (OF)	N/A	NW	-	-	-	-	-	-
232	Cruzeiro do Sul/RS	29°32’23”S52 °00’22”W	2016	*Sida rhombifolia* (OF)	N/A	NW	-	-	-	-	-	-
233	Cruzeiro do Sul/RS	29°30’55”S52 °01’08”W	2016	*Phaseolus. Vulgaris* (OF)	N/A	NW	-	-	-	-	-	-
234	Cruzeiro do Sul/RS	29°32’23”S52 °00’11”W	2016	*Bidens* sp. (OF)	N/A	NW	-	-	-	-	-	-
235	Caxias do Sul/RS	29°19’53”S 51°11’14”W	2016	*Solanum lycopersicum* (GH)	N/A	MEAM1	-	-	-	-	-	-

236	Bom Princípio/RS	29°20’49”S51 °20’38”W	2016	*Cucumis sativus* (GH)	N/A	MEAM1	-	-	-	-	-	-
237	Uberlandia/ MG	18°54' 33"S 48°15'38"W	2017	*Capsicum* spp. (FS)	MG0292 61	MED	-	-	-	-	-	-

Due to the high number of samples, just a representative number of sampling sites were analyzed for the presence of endosymbiont (143 out of 237) and nucleotide sequencing (121 out of 237).

Supplementary Table 2: Virus-infected plants associated to different whitefly species

Sampling Site	Target viruses on PCR	Detected Virus	Whitefly species	Host plant species	City/State
**83**	*Torradovirus*	-	MED	*Begonia* spp.	Santa Isabel/SP
**84**	*Torradovirus*	-	MED	*Hydrangea macrophylla*	Santa Isabel/SP
**85**	*Torradovirus*	-	MED	*Begonia* spp.	Guarulhos/SP
**89**	*Torradovirus*	-	MED	*Petunia* spp.	Mogi Mirim/SP
**91**	*Torradovirus*	-	MED	*Euphorbia pulcherrima*	Londrina/PR
**132**	*Crinivirus* and *Begomovirus*	ToCV	*Trialeurodes vaporariorum*	*S. lycopersicum*	Caxias do Sul/RS
**136**	*Crinivirus* and *Begomovirus*	ToCV + ToSRV	MEAM1	*Solanum lycopersicum*	Lins/SP
**185**	*Carlavirus* and *Begomovirus*	CpMMV	MEAM1	*Glycine max*	Casa Branca/SP
**161**	*Torradovirus*	-	MED	*E. pulcherrima*	São Pedro/SP
**162**	*Torradovirus*	-	MED	*E. pulcherrima*	Atibaia/SP
**223**	*Carlavirus* and *Begomovirus*	CpMMV	MEAM1	*G. max*	Luís Eduardo Magalhães/BA

Supplementary Table 3. Primers used for whiteflies identification, endosymbiont screening and virus identification

**Identification of whiteflies, viruses and endosymbionts**	**Targeted gene**	**Primer sequence (5'>3')**	**Tm (°C)**	**Reference**
MEAM1/MED differentiation	microsatellite locus	Bem23F- CGGAGCTTGCGCCTTAGTC<br>Bem23R- CGGCTTTATCATAGCTCTCGT	55	33, 61, 62
Bemisia tabaci identification	mtCOI	C1-J-2195F-<br>TTGATTTTTTGGTCATCCAGAAGT<br>TL2-N-3014R-<br>TCCAATGCACTAATCTGCCATATTA	45	63
Trialeurodes vaporariorum identification	mtCOI	TvapF-<br>GGCATTATTTCTCATCTTATTAGTGCT<br>WfR- GTGAYTAAGRGMTGGYTTATT	45	65
Portiera aleyrodidarum	16S rRNA	F-<br>GCCCGCCGCGCCCCGCGCCCGTCCCGC<br>CGCCCCCGCCCG<br>R- CCGTCAATTCMTTTGAGTTT	60	66
Rickettsia	16S rDNA	F- GCTCAGAACGAACGCTATC<br>R- GAAGGAAAGCATCTCTGC	60	68
Hamiltonella	16S rDNA	F- TGAGTAAAGTCTGGAATCTGG<br>R- AGTTCAAGACCGCAACCTC	60	67
Wolbachia	16S rDNA	F- CGGGGGAAAAATTTATTGCT<br>R- AGCTGTAATACAGAAAGTAAA	55	69
Arsenophonus	23S rDNA	F- CGTTTGATGAATTCATAGTCAAA<br>R- GGTCCTCCAGTTAGTGTTACCCAAC	60	70
Cardinium	16S rDNA	F- GCGGTGTAAAATGAGCGTG<br>R- ACCTMTTCTTAACTCAAGCCT	58	71
Fritschea	23S rDNA	F- GATGCCTTGGCATTGATAGGCGATG<br>AAGGA<br>R- TGGCTCATCATGCAAAAGGCA	60	72

Torradovirus	RNA-dependent RNA polymerase (RdRp) region in RNA1	Torrado1F- GCWGAYTAYTCMAGYTTTGATGG<br>Torrado2R- GGWACWGCMACHAGRTTGTCATC	52	77
	Vp35 and Vp26 in RNA2	Torrado2F- TGGGATGARTGYAATGTKCT<br>Torrado2R- CCWGTCCACCAYTTGCAATT	50	77
CpMMV	Coat protein region	1280F- GGCGTTCCAAAAGCTGCCGAT<br>1696R- GGAGCCACCTTTCCAATCAA	55	50
ToCV	Heat shock protein (HSP70)	HS11F- GG(G/T)TT(A/G)GA(G/T)TT(C/T)GGTACTAC<br>HS12R- CC(G/T)CCACCAAA(A/G)TCGTA	43	76
		ToC5F- GGTTTGGATTTTGGTACTACATTCAGT<br>ToC6R- AAACTGCCTGCATGAAAAGTCTC	60	76
ToSRV	AL1 and AR1 ORFs of DNA-A	1978F- GCATCTGCAGGCCCACATYGTCTTYCCNGT<br>496R- AATACTGCAGGGCTTYCTRTACATRGG	57	75

Supplementary Figure 1. Sampling sites where the facultative endosymbiont *Wolbachia* was identified, sequenced and phylogenetically analysed in whiteflies. *Bemisia tabaci* New World (sampling site 37), *Bemisia tuberculata* (sampling site 34) and *B. tabaci* Mediterranean (sampling sites 104, 108 and 165). Map data: Google Imagery 2018 TerraMetrics.

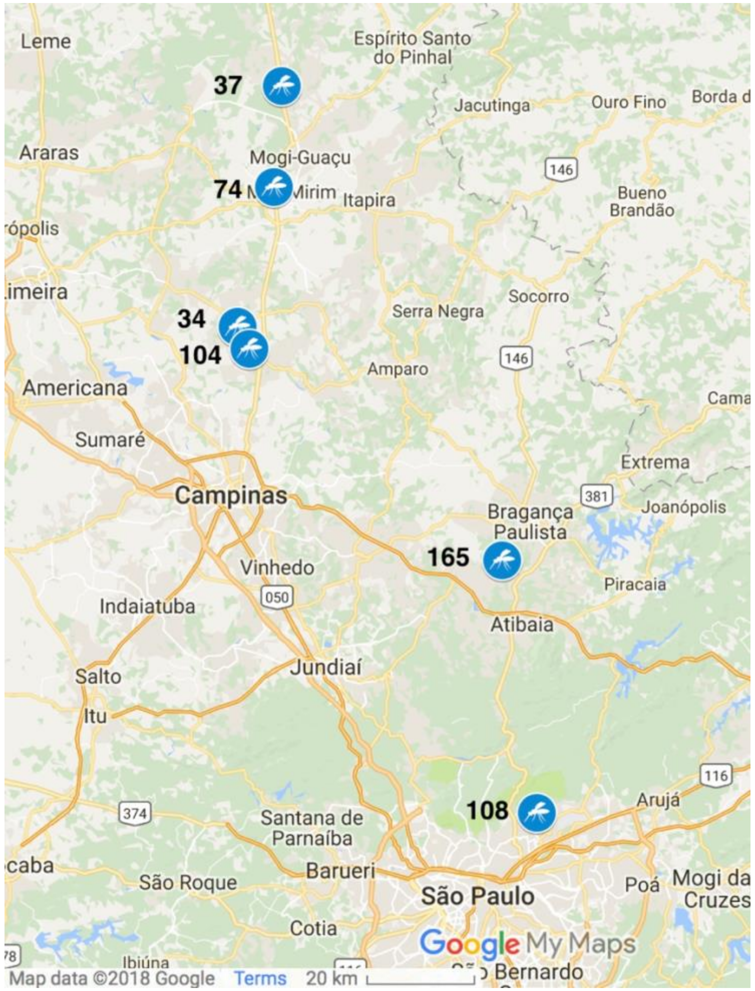

Supplement: Supplementary file 1 — Supplementry Infornation [file 41598_2018_32913_MOESM1_ESM.pdf]
